# Genome-wide association Scan of dental caries in the permanent dentition

**DOI:** 10.1186/1472-6831-12-57

**Published:** 2012-12-21

**Authors:** Xiaojing Wang, John R Shaffer, Zhen Zeng, Ferdouse Begum, Alexandre R Vieira, Jacqueline Noel, Ida Anjomshoaa, Karen T Cuenco, Myoung-Keun Lee, James Beck, Eric Boerwinkle, Marilyn C Cornelis, Frank B Hu, David R Crosslin, Cathy C Laurie, Sarah C Nelson, Kimberly F Doheny, Elizabeth W Pugh, Deborah E Polk, Robert J Weyant, Richard Crout, Daniel W McNeil, Daniel E Weeks, Eleanor Feingold, Mary L Marazita

**Affiliations:** 1 Center for Craniofacial and Dental Genetics, School of Dental Medicine, University of Pittsburgh, Pittsburgh, PA, 15219, USA; 2 Department of Oral Biology, School of Dental Medicine, University of Pittsburgh, Pittsburgh, PA, 15261, USA; 3 Department of Human Genetics, Graduate School of Public Health, University of Pittsburgh, Pittsburgh, PA, 15261, USA; 4 Department of Biostatistics, Graduate School of Public Health, University of Pittsburgh, Pittsburgh, PA, 15261, USA; 5 UNC School of Dentistry, North Carolina Oral Health Institute, Chapel Hill, NC, 27599, USA; 6 IMM Center for Human Genetics and Division of Epidemiology, School of Public Health, University of Texas, Houston, Texas, 77030, USA; 7 Department of Nutrition, Harvard School of Public Health, Boston, Massachusetts, 02115, USA; 8 Department of Biostatistics, University of Washington, Seattle, WA, 98195, USA; 9 Center for Inherited Disease Research, School of Medicine, Johns Hopkins University Baltimore, Baltimore, MD, 21205, USA; 10 Department of Dental Public Health, University of Pittsburgh, School of Dental Medicine, Pittsburgh, PA, 15261, USA; 11 Department of Behavioral and Community Health Sciences, Graduate School of Public Health, University of Pittsburgh, Pittsburgh, PA, 15261, USA; 12 Department of Periodontics, West Virginia University School of Dentistry, Morgantown, WV, 26506, USA; 13 Dental Practice and Rural Health, West Virginia University School of Dentistry, Morgantown, WV, 26506, USA; 14 Department of Psychiatry, School of Medicine, University of Pittsburgh, Pittsburgh, PA, 15213, USA; 15 Clinical and Translational Science Institute, University of Pittsburgh, Pittsburgh, PA, 15213, USA; 16 Department of Pediatric Dentistry, School of Dental Medicine, University of Pittsburgh, Pittsburgh, PA, 15261, USA

**Keywords:** Dental caries, Genetics, Genome wide association, Permanent dentition, Genomics

## Abstract

**Background:**

Over 90% of adults aged 20 years or older with permanent teeth have suffered from dental caries leading to pain, infection, or even tooth loss. Although caries prevalence has decreased over the past decade, there are still about 23% of dentate adults who have untreated carious lesions in the US. Dental caries is a complex disorder affected by both individual susceptibility and environmental factors. Approximately 35-55% of caries phenotypic variation in the permanent dentition is attributable to genes, though few specific caries genes have been identified. Therefore, we conducted the first genome-wide association study (GWAS) to identify genes affecting susceptibility to caries in adults.

**Methods:**

Five independent cohorts were included in this study, totaling more than 7000 participants. For each participant, dental caries was assessed and genetic markers (single nucleotide polymorphisms, SNPs) were genotyped or imputed across the entire genome. Due to the heterogeneity among the five cohorts regarding age, genotyping platform, quality of dental caries assessment, and study design, we first conducted genome-wide association (GWA) analyses on each of the five independent cohorts separately. We then performed three meta-analyses to combine results for: (i) the comparatively younger, Appalachian cohorts (N = 1483) with well-assessed caries phenotype, (ii) the comparatively older, non-Appalachian cohorts (N = 5960) with inferior caries phenotypes, and (iii) all five cohorts (N = 7443). Top ranking genetic loci within and across meta-analyses were scrutinized for biologically plausible roles on caries.

**Results:**

Different sets of genes were nominated across the three meta-analyses, especially between the younger and older age cohorts. In general, we identified several suggestive loci (P-value ≤ 10E-05) within or near genes with plausible biological roles for dental caries, including RPS6KA2 and PTK2B, involved in p38-depenedent MAPK signaling, and RHOU and FZD1, involved in the Wnt signaling cascade. Both of these pathways have been implicated in dental caries. ADMTS3 and ISL1 are involved in tooth development, and TLR2 is involved in immune response to oral pathogens.

**Conclusions:**

As the first GWAS for dental caries in adults, this study nominated several novel caries genes for future study, which may lead to better understanding of cariogenesis, and ultimately, to improved disease predictions, prevention, and/or treatment.

## Background

Dental caries is a common chronic disease that causes pain and disability across all age groups [1]. Untreated caries can lead to pain spread of infection to adjacent tissue, tooth loss, and edentulism (total tooth loss). Caries prevalence increases with age, and by the third decade of life, approximately 91% of dentate adults have experienced dental caries in the US. Although overall caries experience has decreased by about 3.3% over the last decade, this trend is most apparent in younger adults (aged 20–39 years) with higher educational attainment (NHANES surveillance summaries on oral health, 2005). Nevertheless, about 23% of adults have untreated tooth decay, nationwide.

The etiology of dental caries involves a complex interplay of environmental and genetic factors. Heritability analyses have revealed the notable role of genes on caries disease [2-4]. We previously conducted a heritability analysis on dental caries based on 2,600 participants from 740 multi-generational families [5]. For caries in the permanent dentition, we estimated approximately 35-55% of phenotypic variation in disease experience was attributable to genetic factors. Importantly, we also showed that genes affecting susceptibility to caries in the primary dentition partly differ from those in permanent teeth.

Previous studies of the genetics of dental caries have focused mostly on candidate genes. Genes affecting taste preferences (such as taste receptor gene *TAS2R38*) may affect dietary habits, a major known caries risk factor [6]. Other examples are amelogenin (*AMELX*) [7,8] and tuftelin (*TUFT1*) [9], enamel matrix proteins, and *CD14* , an innate immune response gene involved in bacterial pattern-recognition during cariogenesis [10]. In the only genome-wide association study (GWAS) conducted to date on caries [11], a few loci (*ACTN2*, *MTR*, and *EDARADD*, *MPPED2,* and *LPO*) with possible biological roles in susceptibility to caries, although not genome-wide significant, demonstrated suggestive evidence for association with caries phenotypes.

Despite these efforts, few specific genes for dental caries in the permanent dentition have been identified or replicated. Therefore, our goal was to perform genome-wide association scans (GWAS) to identify genetic variants associated with dental caries in permanent dentition in adults. Identification of caries genes will contribute to our understanding of caries etiology, and may lead to preventative interventions and/or treatment strategies for dental caries.

## Methods

### Sample recruitment and data collection

As shown in Table 1, five independent samples were included in this study. 1) The first sample (N = 970) was ascertained through the Center for Oral Health Research in Appalachia (COHRA), an initiative to study the causes of oral health disparities in rural Appalachia. In brief, the sample was drawn from largely rural Appalachian communities in Pennsylvania and West Virginia according to a household-based recruitment protocol requiring at least one biological child–parent pair in order to participate [12]. 2) The second cohort of participants (N = 223, DRDR1) was ascertained through the University of Pittsburgh, School of Dental Medicine Dental Registry and DNA Repository (DRDR). In this ongoing project, every individual that comes to the dental school for treatment is invited to be part of the registry [13]. These samples together with the COHRA sample were included as part of GENEVA dental caries project [14]. 3) The third cohort comprises an additional 290 participants subsequently accepted into the DRDR (DRDR2), with similar demographic characteristics as DRDR1. 4) The fourth cohort (N = 4230) was from the Atherosclerosis Risk in Communities (ARIC) Study, which was designed to investigate the etiology and natural history of atherosclerosis [15]. The Dental ARIC, an ancillary project supported by the National Institute of Dental and Craniofacial Research (NIDCR), was conducted at the fourth visit between 1996 and 1998 [16]. 5) The fifth cohort was from a nested case–control of type 2 diabetes samples within the Health Professionals Follow-up Study [17,18] (HPFS; N = 1730), a prospective on-going project targeting male health professionals aged between 40 and 75 years in the US. Parti cipants particularly involved in our project were recruited in the middle or late 1990s for both ARIC and HPFS, whereas for COHRA and the two DRDR cohorts, samples were brought in on or after 2005. Recruiting for all five sample cohorts was not based on participants’ dental caries status. Written informed consent was obtained from all participants at each individual project. All study procedures were reviewed and approved by the Institutional Review Boards at universities at each site (Federal Wide Assurance (FWA) # for GENEVA dental caries project: FWA00006790; ARIC project: FWA00004801 and HPFS-T2D: FWA00000484).

**Table 1:** Description of the five cohorts

Cohort Description^*^	***COHRA***	***DRDR1***	***DRDR2***	***ARIC***	***HPFS***
*PI*	Marazita	Vieira & Marazita	Vieira	Boerwinkle	Hu
*Sample Size*^†^	970	223	290	4230	1730 (male)
*Age*^**§**^	34.3 ± 9.4	41.9 ± 16.9	41.8 ± 17.9	63.1 ± 5.6	65.2 ± 8.4
*Age Range*	17-67	17-84	17-89	53-75	49-83
*Caries Prevalence (%)*	95.3	94.5	97.7	99.5	98.7
*Caries Phenotype*^**¶**^	DMFS	DMFS	DMFS	Proportion DFS	Caries Severity
*Genotyping Platform*	Illumina 610-Quad	Illumina 610-Quad	Illumina 610-Quad	Affymetrix 6.0	Affymetrix 6.0
Genotyping Cente^**^	CIDR	CIDR	PITT-GPCL	BICGA	BICGA
*Imputed data*	Y	Y	N	Y	N

* COHRA: Center for Oral Health Research in Appalachia cohort.

DRDR1: Dental Registry and DNA Repository cohort phase 1.

DRDR2: Dental Registry and DNA Repository cohort phase 2.

ARIC: Atherosclerosis Risk in Community cohort.

HPFS: Health Professionals Follow-Up Study cohort.

† All summary statistics and subsequent analyses only include genotyped non-Hispanic Whites.

**§** Mean ± SD.

**¶** DMFS: Decayed, Missing due to Decay, or Filled tooth surfaces.

Proportion DFS: Decayed or Filled tooth surfaces / total surfaces at risk.

Caries severity: total # of caries coded as 0, 1; 2–4; 5–9 and 10 or more.

** CIDR: the Johns-Hopkins Center for Inherited Disease Research;

PITT-GPCL: Genomics and Proteomics Core Laboratories at University of Pittsburgh.

BICGA: Broad Institute Center for Genotyping and Analysis at Harvard and MIT.

### Caries Phenotype assessment

For COHRA, dental caries of permanent teeth was assessed by dentists or dental hygienists via visual inspection. Data for DRDR1 and DRDR2 were extracted from evaluations done by dentists. Examiners across all sites were calibrated periodically. Each tooth surface was scored as sound, decayed, filled, missing due to decay, or missing due to reasons other than decay, in accordance with the World Health Organization recommended scale and in accordance with the NIH/NIDCR-approved protocol for assessing dental caries for research purposes [12,19]. This method of caries assessment is compatible with the Phen-X Toolkit ( http://www.phenxtoolkit.org) to facilitate combining data across studies, and the National Center for Health Statistics Dental Examiners Procedures Manual (See Section 4.9.1.3). Third molars were excluded from caries assessment. Edentulous individuals were recruited into the study but were excluded from caries assessment and follow-up analysis. The phenotype, DMFS, used in GWAS analysis represents the count of decayed, missing due to decay, or filled (restored) tooth surfaces across an individual’s permanent dentition.

Caries assessment in the ARIC cohort was similar to the approach indicated above, except that no distinction was made between teeth that were missing due to decay or missing due to another reason. Thus, the DFS (decayed or filled tooth surface) phenotype was available for this dataset. In order to account for the variation of total number of teeth at risk among this older sample of individuals, we created a new phenotype where the proportion of DFS equals to the original DFS counts divided by the total number of tooth surfaces at risk.

In the HPFS cohort, caries was assessed by self-reported questionnaires. Baseline caries measurement collected in 1996 was used in our analysis. In general, data was collected on the total number of cavities in permanent teeth. The response to this question was an ordered categorical variable representing different levels of caries severity (no cavity, 1 affected tooth, 2–4, 5–9, and 10 or more affected teeth).

As reported previously [6,12], both inter- and intra-examiner concordances of caries assessments were high in the COHRA cohort. However this calibration process was not available for other cohorts, either because such design was not part of the original study (DRDR1 and DRDR2), or the caries phenotype collection was of a side interest (ARIC), or the caries assessment was simply based on self-reported information from questionnaire (HPFS).

### Genotyping, quality assurance, and imputation

As part of GENEVA dental caries project, genotyping for COHRA and DRDR1 samples was carried out on behalf of the GENEVA consortium by the Johns-Hopkins Center for Inherited Disease Research (CIDR) through a National Institutes of Health contract. Genotyping of these cohorts was performed using the Illumina Human610-Quadv1_B BeadChip (Illumina, San Diego, CA, USA). Additional details are available at the National Center for Biotechnology Information database of Genotype and Phenotypes (dbGaP, http://www.ncbi.nlm.nih.gov/sites/entrez?db=gap, study accession designation phs000095.v1.p1). The DRDR2 cohort was genotyped at the University of Pittsburgh Genomics and Proteomics Core Laboratory using the same Illumina Human610-Quad chip. Genotyping for both ARIC and HPFS cohorts was performed at the Broad Institute of MIT and Harvard’s Center for Genotyping and Analysis using the Affymetrix 6.0 SNP array (Affymetrix, Santa Clara, CA, USA) and the Birdseed calling algorithm. Additional details are available at dbGaP (study accession designations phs000090.v1.p1 for ARIC and phs000091.v2.p1 for HPFS)

Genotype data for all cohorts except DRDR2 went through an extensive process of cleaning, imputation, and quality assurance, performed by the GENEVA consortium Coordinating Center at the University of Washington [14,20,21]. The entire cleaning procedure included but was not limited to, checks for gender identity, chromosomal anomalies, sample relatedness, population structure, missing call rates, plate effects, Mendelian errors, duplicate discordance, etc. Detailed cleaning reports are publicly available for each study at the above referenced dbGaP resource. The data cleaning and quality control for DRDR2 genotypes were conducted by our own team using similar procedures as above.

Genotype imputation (i.e., inferring unobserved genotypes based on observed ones from a reference sample with similar genetic background) was performed by the GENEVA coordinating center for three cohorts (COHRA, DRDR1 and ARIC). Imputed data were released for all successfully imputed SNPs (approximately 1.4 million) using subjects from a HapMap Phase III reference panel (genetically-determined European ancestry, CEU sample) and BEAGLE software [22]. Quality metrics were provided for each imputed SNP that were further used in analysis for filtering imputation results on a per-SNP level. Imputed genotypes are provided as the probability of each of the three genotype states, reflecting the level of certainty in the genotype prediction. These probabilities were directly incorporated into downstream statistical analyses within PLINK, rather than taking the most likely imputed genotype. For detailed description of this imputation procedure and follow-up quality control, please refer to the report available on dbGaP.

### Statistical analysis

Genome-wide association scans were limited to self-reported non-Hispanic Whites, which comprised the majority of samples in our study. This was to minimize the risk of inflated type I error caused by population stratification and to avoid reduction in power due to possible genetic heterogeneity. Before analysis, principal component analysis (PCA) based on independent autosomal SNPs was applied to verify the self-reported race variable against the DNA evidence. Hapmap controls (CEU, YRI, CHB, JPT) were used as reference. High concordance between self-reported race and genetically-determined ancestry was observed across all cohorts. The very rare outliers were excluded in further analysis. For the COHRA sample, which included participants of all ages, statistical analysis was limited to permanent teeth in individuals 17 years or older. All participants in the other cohorts were adults, and therefore were included in analysis.

All GWAS scans were performed in PLINK ( http://pngu.mgh.harvard.edu/~purcell/plink) [23] using linear regression (−−linear option) while adjusting for age and sex as covariates. The above analyses were performed separately in each cohort with genotyped data and imputed data if available (COHRA, DRDR1 and HPFS). Before analysis, HWE (P-value ≤ 10E-4) and minor allele frequency (MAF ≤ 0.02) filters were applied to exclude outlier or rare SNPs. Next, we combined the GWAS association results from each study by performing meta-analysis in METAL ( http://www.sph.umich.edu/csg/abecasis/Metal/) [24] using its weighted Z-score method based on sample size, P-value and direction of effect in each study (fixed effect model). Due to the differences in age, birth cohort, demography, genotyping platform, and quality of dental caries assessment, as well as possible genetic heterogeneity among our cohorts, we performed three meta-analyses: 1) Meta 1 (COHRA, DRDR1, and DRDR2): we combined these three cohorts because they were each comprised of comparatively younger individuals from Appalachia. In addition, they were genotyped on the same Illumina chip, and have the most informative caries DMFS phenotype; 2) Meta 2 (ARIC and HPFS): we combined these two cohorts because they were both genotyped using Affymerix 6.0 chip and they both included comparatively older participants (all samples ≥49 years) with poorer quality dental caries assessments; 3) Meta 3 (all five cohorts combined).

We explored all signals with “suggestive significance” (P-value ≤ 10E-5) using several online bioinformatics tools and databases, such as SCAN ( http://www.scandb.org/) [25], and WGAViewer ( http://compute1.lsrc.duke.edu/softwares/WGAViewer/) [26]. This step was crucial and based on the assumption that associated SNPs, which may not themselves be causal, were in LD with the causal variant nearby. Moreover, it is currently unknown where a causal variant may be located with respect to the gene it affects, although cis-acting (i.e., physically proximal) variants are widely believed to be important. Therefore, for every SNP meeting suggestive significance, we explored whether any nearby genes had known biological functions relevant to cariogenesis. The calculation of genomic inflation factor, lambda, and the generation of Quantile-Quantile plots were conducted in the R statistical package (R Foundation for Statistical Computing, Vienna, AU). Manhattan plots were created using Haploview [27]. Regional visualization of GWAS top signals were produced using LocusZoom ( http://csg.sph.umich.edu/locuszoom/) [28]. We also generated genotype intensity plots (i.e. cluster plots) for genotyped SNPs within top signals to verify high-quality genotype calling. Because over 95% of our samples were unrelated individuals, we did not adjust analysis for family relatedness, but closely monitored evidence of genomic inflation.

## Results

Table 1 shows descriptive characteristics of the five cohorts used in our study. ARIC and HPFS were the two largest cohorts containing comparatively older participants aged 49 years or greater. The mean ages of these cohorts were more than 20 years greater than those from the other three cohorts. The difference of birth year is even larger between two older and three younger cohorts because subjects in ARIC and HPFS were ascertained almost 10 years earlier. The HPFS cohort included only males. The DRDR1 and DRDR2 cohorts were similar. Caries prevalence was extremely high (94.5-99.5%) for all of our five cohorts, substantially higher than that reported by NHANES in 2005 (86.8-96.3%) for corresponding age groups.

Different methods of caries assessment were performed across the five cohorts (Table 1). Tooth surface-level caries assessment was performed for COHRA, DRDR1 and DRDR2, by intra-oral examination, from which DMFS index was generated. DMFS index is the count of carious surfaces across the dentition, and is the most widely used measure of dental caries experience along with DMFT (index by tooth). Caries measurements in the other two cohorts were different and presumably less complete from above. In ARIC, data on teeth missing due to decay were not collected, and therefore the DMFS index could not be generated. Instead we used the proportion DFS as our caries phenotype, which measures caries experience with respect to the number of tooth surfaces for which we have data (as opposed to the full permanent dentition, as in DMFS). In HPFS, dental caries was assessed as a self-reported categorical variable representing approximate number of carious lesions at tooth level.

**Figure 1:**
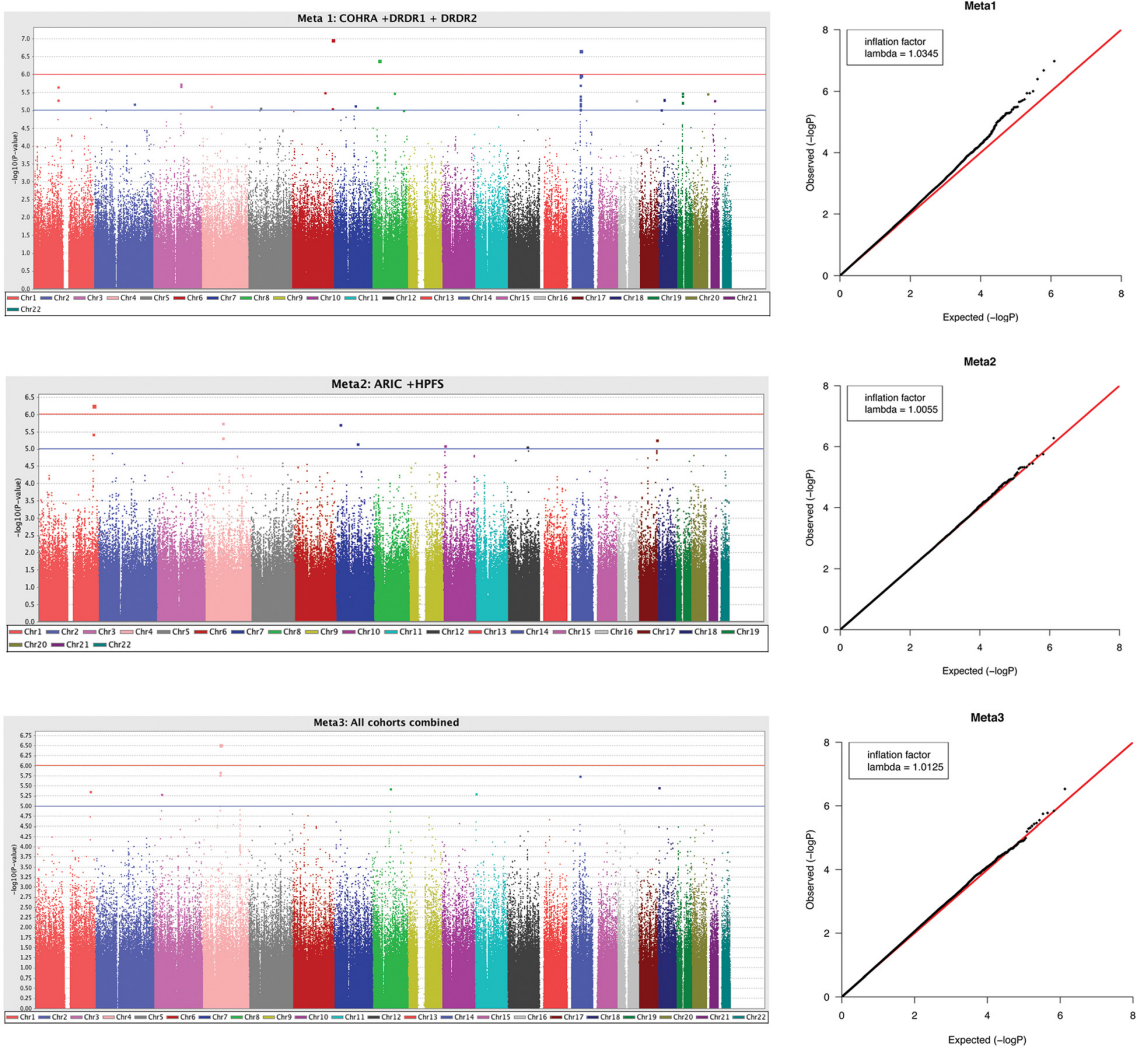
**GWAS results in three Meta-Analyses: Manhattan and Q-Q plots.** All P-values are negative log_10_ transformed. Each point represents a genotyped or imputed (whenever available) SNP marker.

Figure 1 shows Manhattan plots for the three meta-analyses. No association signals passed the genome-wide significance threshold (i.e., marginal P-value ≤ 5.0 × 10^-8^). The genomic inflation factor, λ, was 1.0345, 1.0055 and 1.0125 for three meta-analyses, respectively, indicating negligible P-value inflation. We investigated the genes (and possible biological functions) at or near SNPs with suggestive P-values (i.e., P-value ≤ 10E-5) in each meta-analysis, and compared common genetic signals across meta-analyses.

### Top Signals within each meta-analysis (P-values ≤ 10E-7)

**Figure 2:**
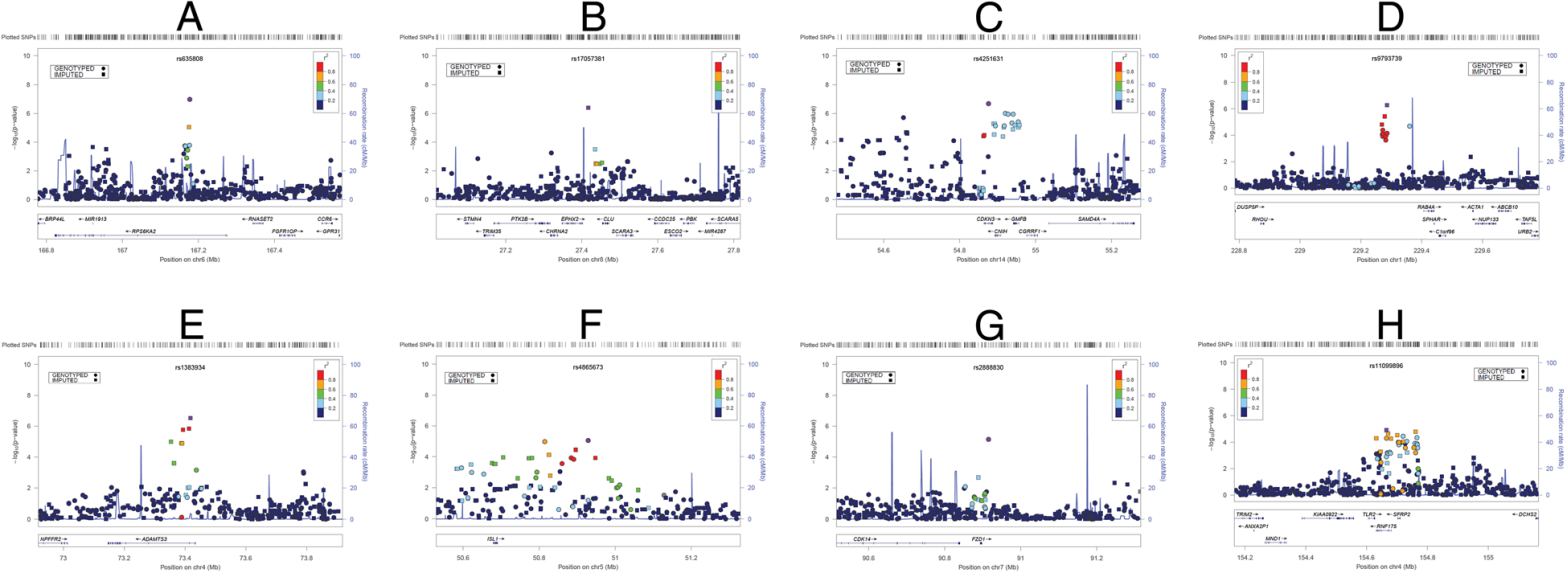
**Regional plots of P-values at top loci in meta-analyses.** Negative log_10_ transformed P-values and physical positions for SNPs in the region are shown. Colors indicate linkage disequilibrium between the index SNP (colored in purple) and other SNPs based on HapMap CEU data. The rug plot indicates regional SNP density. The recombination rate overlay is based on HapMap CEU data. Gene positions and directions of transcription are annotated based on hg19/1000 Genomes Nov 2010 release.

Altogether, there were 5 regions identified in our study where at least one SNP achieved this level of significance: three from Meta 1 and one each from Meta 2 and 3 (Table 2). The SNP exhibiting the strongest evidence of association in Meta 1 was rs635808 on chromosome 6 (P-value = 1.06 × 10^-7^) located in the intronic region of *RPS6KA2* (Figure 2A, Additional file 1: Table S1). This gene encodes an enzyme from the RSK (ribosomal S6 kinase) family, which is capable of phosphorylating various substrates, including members of the mitogen-activated kinase (MAPK) signaling pathway. It has been previously reported that the activation of MAPK pathway (through p38 phosphorylation) plays pivotal role in inflammatory cytokine and chemokine gene regulation and thus it is involved in oral-related diseases such as dental caries [29], caries-induced pulpitis [30], chronic oral pain and periodontal disease. 

**Table 2:** Effect size and P-values for top SNPs in three meta-analyses^*^

**Gene/SNPs**	**Chr**	**Base Pair position**	**Data Status**^†^	**Effect Size**^**§**^					**P-value (Meta 1)**	**P-value (Meta 2)**	**P-value (Meta 3)**
				***COHRA***	***DRDR1***	***DRDR2***	***ARIC***	***HPFS***			
**RHOU**											
rs3936161	1	227336163	Illumina	−1.95	7.17	--	−0.70	--	0.721	1.55E-05	6.76E-05
rs12072775	1	227339176	Affymetrix	−1.94	7.17	--	−0.71	0.007	0.725	4.23E-05	0.001
rs9287022	1	227344972	Imputed	−2.09	7.19	--	−0.61	--	0.673	3.79E-06	1.86E-05
rs9793739	1	227352481	Imputed	−2.08	7.88	--	−0.75	--	0.721	**5.27E-07**	4.28E-06
rs2988738	1	227427128	Affymetrix	0.67	9.55	--	−1.75	−0.15	0.567	2.02E-05	0.002
**ADAMTS3**											
rs788919	4	73572758	Illumina	−1.40	−4.14	−3.56	−0.47	--	0.026	1.36E-04	1.02E-05
rs4694123	4	73606652	Illumina	−1.31	−2.94	−3.89	−0.46	--	0.038	1.18E-04	1.26E-05
rs10805050	4	73612147	Illumina	1.01	2.93	2.50	0.42	--	0.093	4.88E-06	1.68E-06
rs788911	4	73632087	Illumina	0.99	2.54	3.29	0.38	--	0.084	4.77E-06	1.46E-06
rs1383934	4	73636388	Illumina	1.19	3.20	3.70	0.64	--	0.046	1.77E-06	**2.96E-07**
**RPS6K2**											
rs505982	6	167095386	Imputed	3.66	8.52	--	−0.35	--	8.93E-06	0.859	0.025
rs635808	6	167097412	Illumina	−4.21	−7.53	−8.30	0.44	--	**1.06E-07**	0.898	0.010
**PTK2B**											
rs17057381	8	27416801	Affymetrix	16.39	28.98	--	0.47	−0.03	**4.02E-07**	0.267	0.764
**CNIH**											
rs1953743	14	53722229	Both	−3.08	−6.55	−6.87	0.01	0.04	1.98E-06	0.371	0.027
rs4251631	14	53945934	Illumina	−3.78	−6.32	−10.02	−0.35	--	**2.13E-07**	0.013	1.80E-06
rs11850320	14	53990173	Illumina	−4.57	−7.93	−6.78	−0.33	--	**9.92E-07**	0.177	0.0003
rs7150062	14	53997400	Both	4.52	7.93	6.78	0.40	0.01	1.15E-06	0.295	0.001
rs7143579	14	54010435	Illumina	−4.42	−7.15	−7.89	−0.42	--	1.16E-06	0.137	0.0002

* Summarizes genes/regions containing at least one SNP with significant P-values ≤ 10E-7 (bolded); Listed are the first five most significant SNPs if more than five SNPs observed at the corresponding region;

† Illumina/Affymetrix/Both: SNP was genotyped in Illumina 610Quad/Affymetrix 6.0/both chips respectively;

Imputed: SNP data was generated by imputation only. “--" indicating the corresponding SNP was not genotyped in DRDR2 or

HPFS;

**§** Effect size can be directly compared ONLY among Meta 1 cohorts (COHRA, DRDR1 and DRDR2).

Another suggestive signal observed in Meta 1 was rs17057381 (P-value = 4.02 × 10^-7^) on chromosome 8. Within a ±100 kb region, there are five genes including *PTK2B*. No direct evidence implicates these genes in cariogenesis; however, previous studies have shown that *PTK2B* mediates the p38-dependent MAPK pathway [31,32] and is important for oral disorders including dental caries. (Figure 2B)

The third suggestive signal observed in Meta 1 was a broad region of association on chromosome 14 (Figure 2C; top SNP was rs4251631, P-value = 2.13 × 10^-7^). Multiple low LD SNPs (in reference to rs4251631) demonstrated suggestive significance and four of them were among the top SNPs in Meta 3 (P-values between 8.17 × 10-5 and 1.80 × 10-6). The association signal is centered over a region of low recombination harboring 4 genes, *CDKN3, CNIH, GMFB* and *CGRRF1* (none of which have known or biologically plausible roles in dental caries). The association signal extends 500 kb upstream to the 5’ untranslated region of *BMP4* gene. Bone morphogenetic proteins are important for regeneration/repair of the dentin-pulp complex after cariogenic injury [33], and *BMP4*, in particular, has been shown to initiate and regulate repair of carious tissue [34,35].

In Meta 2 we observed a suggestive signal on chromosome 1 (rs9793739, P-value = 5.27 × 10^-7^). No relevant information with caries was found for genes near this SNP except that about 400 kb upstream of the top hit, was the RHOU gene (the closest hit, Figure 2D), a member of the Rho family of GTPases. Evidence suggests that GTPases act as key mediators of the Wnt signaling cascade [36], a pathway that is well-known for its role in regulating tooth morphology during tooth development [37]. In 2001, Tao *et al.* showed in mice the possible role of *RHOU* in the regulation of cell morphology and proliferation through the Wnt1 signaling pathway [38]. Though biologically plausible, it is currently unknown whether RHOU is involved in genetic susceptibility to dental caries.

In Meta 3 we observed a suggestive association with rs1383934 (P-value = 2.96 × 10^-7^). This SNP is located on chromosome 4 in the intronic region of *ADAMTS3* (Figure 2E), which is highly expressed during tooth development in the dental papilla in mice [39]. The role of *ADAMTS3* in cariogenesis is unknown; however, given its role in tooth development in mouse, it is plausible that this gene affects susceptibility to dental caries.

### Other interesting signals (P-values ≤ 10E-5)

In Meta 1 we also observed suggestive association for a 400 kb region on chromosome 5 including the *ISL1* gene (rs4865673, P-value = 8.73 × 10^-6^, Figure 2F). In mice, this gene is exclusively expressed in epithelial cells of developing incisors, and is a crucial regulator of jaw and tooth development [40], suggesting a possible mechanism through which *ISL1* may affect susceptibility to dental caries.

For Meta 2, we also observed suggestive association with the gene *FZD1* on chromosome 7 (rs2888830, P-value = 7.01 × 10^-6^, Figure 2G). As receptor of Wnt family signaling molecules, FZD1 is responsible for activating intracellular signals for Wnt pathways for tooth initiation (eruption) [41].

In Meta 3, we observed suggestive association with the gene *TLR2* on chromosome 4 (rs11099896, P-value = 1.24 × 10^-5^, Figure 2H). *TLR2* is involved in the immune response against cariogenesis; the gene-coded receptor is expressed on the cell surface of odontoblasts. During cariogenesis, the receptor recognizes oral bacterial and triggers the immune defense system [42]. In both dentin [43] and dental pulp [44], similar mechanisms were observed.

### Cross-Meta-analysis signals

**Figure 3:**
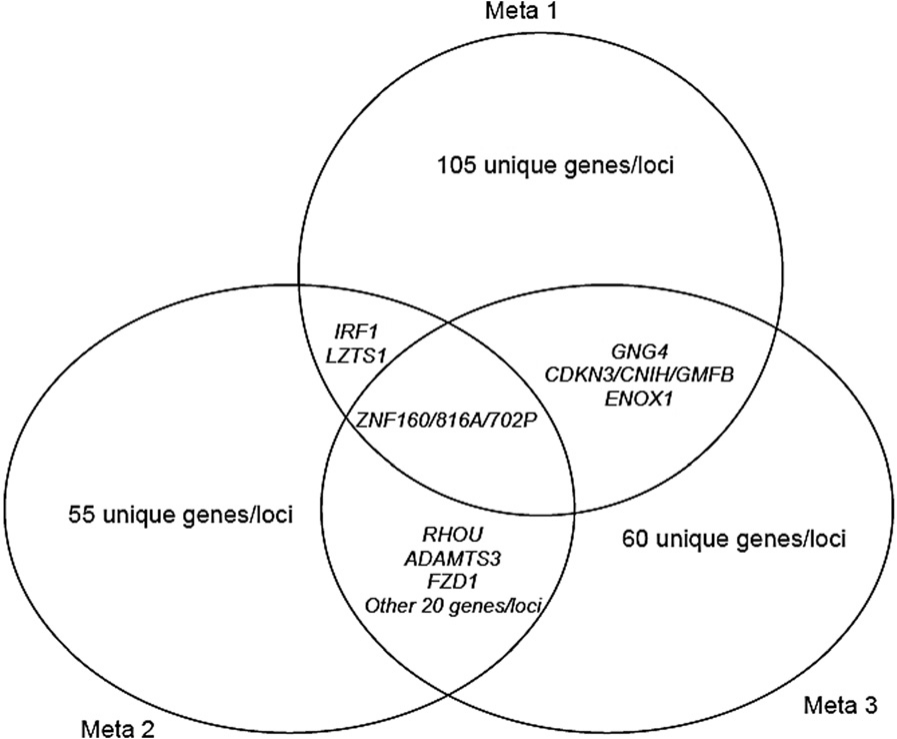
Venn diagram summarizing common Genes (on or near SNPs with P-value ≤ 10E-5) cross meta-analyses.

Shared signals were observed across meta-analyses including associations of common SNPs and common regions (i.e., within 100 kb) in two or more meta-cohorts. There were 29 loci that exhibited suggestive association across meta-analyses (See Figure 3 and Additional file 1: Table S3). Besides genes (such as RHOU, ADAMTS3, CDKN3/CNIH/GMFB, FZD, etc.) which had been highlighted in individual meta-analysis, this list also includes *ZNF160* on chromosome 19 (rs10405102, P-value = 3.02 × 10^-5^ in Meta 1; rs9967593 and rs1650966, P-value = 2.23 × 10^-5^ and 2.22 × 10^-5^ respectively in Meta 2; rs2288421, P-value = 5.96 × 10^-5^ in Meta 3), which represses TLR4 [45], another odontoblast cell-surface receptor that recognizes oral pathogens to mediate immune response [46].

## Discussion

We performed the first GWAS for dental caries in the permanent dentition in adults, which complements earlier scans for childhood caries [11], tooth eruption [47] and the whole genome linage scans for caries using family data [48]. Though we did not observe any genetic associations meeting genome-wide significance, we did nominate several statistically suggestive loci with plausible biological roles in dental caries. Specifically we nominated *RPS6KA2* and *PTK2B* involved in p38-dependent MAPK signaling; *RHOU* and *FZD1* involved in Wnt signaling cascade. Both of these pathways have been implicated in dental caries. *ADMTS3* and *ISL1* are involved in tooth development; and *TLR2* is involved in immune response to oral pathogens.

Our study investigated the genetics of dental caries separately in our younger Appalachian cohorts and comparatively older non-Appalachia cohorts. Comparing the ARIC and HPFS cohorts versus the other three Appalachian ones, the mean age difference is over 20 years and the participants in older cohorts were ascertained about 10 years earlier. In other words, subjects were born 30 years earlier, on average, in ARIC and HPFS. We speculate that this birth cohort effect may serve as a surrogate for unmeasured life history variables that differ between the Appalachian and non-Appalachian cohorts. For instance, water and tooth paste fluoridation was introduced between the 1950s and 1970s in the US. For participants in ARIC and HPFS studies, the majority had little exposure to sources of fluoride in their first 20 to 30 years of life. In comparison, the majority of COHRA, DRDR1, and DRDR2 participants had fluoride exposure throughout their entire lives. Given the protective role of fluoride on dental caries, and the likely involvement of gene-by-fluoride interactions, we speculate that fluoride exposure may account for some of the genetic heterogeneity between Meta 1 and Meta 2. Other unknown factors that differ between cohorts may have a similar effect.

This study benefits from several strengths including a large sample size of 7,200 participants, quality genotyping and imputation data generated by CIDR, Broad CGA and the GENEVA coordinating center, and carefully-designed meta-analyses assessing genetic effects within and across multiple cohorts. However, several limitations warrant further discussion. First, we did not replicate genetic association with any genes implicated in the previous GWAS of childhood dental caries. This is perhaps because the current analysis studied a different dentition type (permanent *vs*. primary teeth). In addition, we achieved lower performances in larger cohorts. For example, although Meta 2 had four times larger sample size than Meta 1, in Meta 2 we observed fewer suggestive genetic signals than analysis in Meta 1 (141 *vs.* 222 and 10 *vs.* 41 SNPs of P-values ≤ 10E-5 and 10E-6 respectively). Possible explanations include the poorer quality assessment of caries, the imbalance in the sex ratio, and the advanced age of participants for whom the cumulative environmental assault across decades may have greatly overshadowed genetic effects. Furthermore, during the analysis on HPFS case–control cohort of type 2 diabetes, we failed to adjust the diabetes status variable due to the IRB restriction. There existed evidence showing that individuals with type 2 diabetes may exhibit poorer oral health [49]. However, the definite answer for association between dental caries and type 2 diabetic status remains uncertain [50,51].

## Conclusions

We designed and performed the first genome-wide association study for dental caries in the permanent dentition in adults. The GWAS analyses were first conducted in each of five independent cohorts; three meta-analyses were subsequently performed on part or all data from over 7000 combined samples. Although we did not observe any genetic associations meeting genome-wide significance, we identified a few loci that demonstrated both the suggestive P-values and the biologically relevant functions for dental caries. Of note, several of these nominated genes may be involved in common signaling pathways.

## Competing interests

The authors declare that they have no competing interests.

## Authors’ contributions

XW, JRS, EF, DEW, MLM conceived and designed this study; XW analyzed the data; XW and JRS wrote the manuscript; XW, JRS, ZZ, FB, EF, DEW and MLM managed, cleaned and quality checked the data together with other co-authors from the coordinating center at University of West Virginia, University of Washington, CIDR, ARIC and HPFS collaborators ; XW, JRS, EF, KTC, MKL, DEW and MLM interpreted the results; XW, JRS, ZZ, FB, ARV, KTC, MKL, DEP, RJW, DEW and MLM read, revised and approved the manuscript. All authors read and approved the final manuscript.

## Pre-publication history

The pre-publication history for this paper can be accessed here:

http://www.biomedcentral.com/1472-6831/12/57/prepub

## Supplementary Material

Additional file 1 **SNPs with P-value ≤ 10E-5 in Meta 1, Meta 2 and Meta 3.** This files contains 3 tables (Supplement Table 1A, 1B and 1C), each of which shows the top-hit SNPs (P-value ≤ 10E-5 as cut-off) and other corresponding information from the three meta-analyses (meta 1, meta2 and meta 3) respectively. Click here for file
