# Supplementary material for: Genome-wide association Scan of dental caries in the permanent dentition
Source: BMC Oral Health. 2012 Dec 21;12:57. doi: 10.1186/1472-6831-12-57 (PMC3574042; doi:10.1186/1472-6831-12-57)
Supplement: Additional file 1 — SNPs with P-value ≤ 10E-5 in Meta 1, Meta 2 and Meta 3. This files contains 3 tables (Supplement Table 1A, 1B and 1C), each of which shows the top-hit SNPs (P-value ≤ 10E-5 as cut-off) and other corresponding information from the three meta-analyses (meta 1, meta2 and meta 3) respectively. [file 1472-6831-12-57-S1.docx]

Supplement Table 1A: SNPs with P-value ≤ 10E-5 in Meta 1

| **Chr** | **SNP** | **rank** | **P-value** | **position** | **alleles** | **gene** |
| --- | --- | --- | --- | --- | --- | --- |
| 1 | rs17025313 | 141 | 6.57E-05 | 110340449 | G/T | AHCYL1 |
| 1 | rs720917 | 215 | 9.91E-05 | 110344547 | A/G | AHCYL1 |
| 1 | rs11102043 | 151 | 6.95E-05 | 110318919 | C/T | CSF1 |
| 1 | rs526201 | 212 | 9.79E-05 | 223846789 | C/T | ENAH |
| 1 | rs4971195 | 119 | 5.24E-05 | 155701031 | A/G | ETV3 |
| 1 | rs6427380 | 110 | 4.84E-05 | 155712519 | C/T | ETV3 |
| 1 | rs10518536 | 198 | 9.44E-05 | 93754538 | A/G | FNBP1L |
| 1 | rs508208 | 51 | 1.66E-05 | 233794439 | A/G | GNG4 |
| 1 | rs10863852 | 123 | 5.35E-05 | 208964372 | A/C | KCNH1 |
| 1 | rs1934620 | 121 | 5.31E-05 | 208964886 | A/G | KCNH1 |
| 1 | rs10863853 | 133 | 6.15E-05 | 208966802 | A/G | KCNH1 |
| 1 | rs12027143 | 128 | 5.90E-05 | 99129105 | A/G | PAP2D |
| 1 | rs764051 | 176 | 7.95E-05 | 99129546 | C/T | PAP2D |
| 1 | rs12030278 | 53 | 1.79E-05 | 99132563 | A/G | PAP2D |
| 1 | rs1329465 | 143 | 6.61E-05 | 99155952 | C/T | PAP2D |
| 1 | rs1571500 | 10 | 2.22E-06 | 99158881 | C/T | PAP2D |
| 1 | rs11166135 | 21 | 5.18E-06 | 99121424 | A/G | SNX7 |
| 2 | rs1079204 | 84 | 3.59E-05 | 218838758 | A/G | AAMP |
| 2 | rs2292549 | 90 | 3.85E-05 | 218836750 | A/G | AAMP |
| 2 | rs6735139 | 172 | 7.57E-05 | 73584979 | C/G | ALMS1 |
| 2 | rs7600989 | 93 | 3.90E-05 | 218827379 | G/T | ARPC2 |
| 2 | rs2069213 | 66 | 2.55E-05 | 32596588 | C/T | BIRC6 |
| 2 | rs1567869 | 98 | 4.16E-05 | 218834980 | A/G | GPBAR1 |
| 2 | rs1263625 | 87 | 3.68E-05 | 207685878 | A/G | KLF7 |
| 2 | rs10496012 | 72 | 2.97E-05 | 53117375 | C/T | LOC129656 |
| 2 | rs10929113 | 195 | 8.96E-05 | 235837030 | C/T | LOC642692 |
| 2 | rs11899345 | 210 | 9.78E-05 | 227102137 | A/G | LOC646736 |
| 2 | rs11904820 | 209 | 9.75E-05 | 227111060 | A/G | LOC646736 |
| 2 | rs16867099 | 111 | 4.88E-05 | 227156161 | A/C | LOC646736 |
| 2 | rs876887 | 205 | 9.66E-05 | 134793655 | A/G | MGAT5 |
| 2 | rs11674550 | 214 | 9.88E-05 | 134798914 | A/G | MGAT5 |
| 2 | rs17568791 | 42 | 1.03E-05 | 50874992 | C/T | NRXN1 |
| 2 | rs1017697 | 135 | 6.31E-05 | 218878706 | A/G | PNKD |
| 2 | rs2014597 | 132 | 6.14E-05 | 218879706 | C/T | PNKD |
| 2 | rs6708662 | 152 | 7.06E-05 | 218896913 | C/T | PNKD |
| 2 | rs10192690 | 116 | 5.20E-05 | 218898957 | A/G | PNKD |
| 2 | rs4438497 | 28 | 6.58E-06 | 166885605 | C/T | SCN9A |
| 3 | rs13093350 | 62 | 2.42E-05 | 114475753 | C/T | BOC |
| 3 | rs775227 | 9 | 2.14E-06 | 114477764 | A/C | BOC |
| 3 | rs808951 | 112 | 5.00E-05 | 114484840 | C/T | BOC |
| 3 | rs13064411 | 46 | 1.26E-05 | 114529330 | A/G | BOC |
| 3 | rs6442772 | 196 | 9.20E-05 | 3009425 | C/T | CNTN4 |
| 3 | rs2333035 | 99 | 4.38E-05 | 126282090 | A/G | HEG1 |
| 3 | rs7617001 | 60 | 2.12E-05 | 57024834 | C/T | LOC100128209 |
| 3 | rs9826009 | 68 | 2.74E-05 | 57028468 | C/T | LOC100128209 |
| 3 | rs11922193 | 164 | 7.39E-05 | 57034410 | A/C | LOC100128209 |
| 3 | rs17006578 | 127 | 5.80E-05 | 70017021 | A/G | MITF MITF MITF |
| 3 | rs1996313 | 203 | 9.51E-05 | 193050583 | G/T | PYDC2 |
| 3 | rs13074313 | 74 | 3.06E-05 | 114756324 | A/C | SIDT1 |
| 3 | rs13092825 | 7 | 1.84E-06 | 114773158 | C/T | SIDT1 |
| 3 | rs17239028 | 89 | 3.85E-05 | 126286481 | G/T | SLC12A8 |
| 4 | rs2631766 | 168 | 7.48E-05 | 8469679 | C/G | ACOX3 |
| 4 | rs954270 | 134 | 6.17E-05 | 8472673 | A/G | ACOX3 |
| 4 | rs4466078 | 33 | 7.61E-06 | 40668284 | A/T | APBB2 |
| 4 | rs2631723 | 206 | 9.67E-05 | 8557240 | A/C | C4orf23 |
| 4 | rs7678192 | 190 | 8.77E-05 | 182431572 | C/T | hCG_2025798 |
| 4 | rs17188642 | 136 | 6.31E-05 | 23714707 | C/T | PPARGC1A |
| 4 | rs1562139 | 102 | 4.59E-05 | 82713010 | C/T | RASGEF1B |
| 4 | rs1947070 | 103 | 4.61E-05 | 82718533 | A/C | RASGEF1B |
| 5 | rs6884406 | 137 | 6.31E-05 | 71221928 | A/G | CARTPT |
| 5 | rs6862930 | 181 | 8.21E-05 | 129480557 | A/G | CHSY3 |
| 5 | rs2190815 | 126 | 5.56E-05 | 129481133 | C/T | CHSY3 |
| 5 | rs13170334 | 118 | 5.22E-05 | 129485129 | G/T | CHSY3 |
| 5 | rs173632 | 216 | 9.92E-05 | 129538096 | A/G | CHSY3 |
| 5 | rs13161611 | 80 | 3.51E-05 | 145261803 | G/T | GRXCR2 |
| 5 | rs13182269 | 88 | 3.70E-05 | 145265282 | A/G | GRXCR2 |
| 5 | rs13169102 | 94 | 3.91E-05 | 145265327 | G/T | GRXCR2 |
| 5 | rs739718 | 178 | 8.20E-05 | 131900972 | A/G | IRF1 |
| 5 | rs2068701 | 43 | 1.04E-05 | 50850389 | A/G | ISL1 |
| 5 | rs6894368 | 169 | 7.49E-05 | 50861586 | C/T | ISL1 |
| 5 | rs4865514 | 82 | 3.53E-05 | 50931560 | G/T | ISL1 |
| 5 | rs4865515 | 85 | 3.63E-05 | 50931572 | A/C | ISL1 |
| 5 | rs4865673 | 35 | 8.73E-06 | 50964567 | A/G | ISL1 |
| 5 | rs4240378 | 179 | 8.20E-05 | 123992963 | A/G | KRT18P16 |
| 5 | rs6596271 | 188 | 8.72E-05 | 135248728 | A/G | LOC153328 |
| 5 | rs12055154 | 161 | 7.19E-05 | 176608029 | A/G | NSD1 |
| 5 | rs28710014 | 187 | 8.65E-05 | 176661423 | A/G | RAB24 |
| 5 | rs10073141 | 147 | 6.85E-05 | 123999397 | A/G | ZNF608 |
| 6 | rs9505270 | 44 | 1.06E-05 | 7688662 | A/G | BMP6 |
| 6 | rs9503233 | 155 | 7.13E-05 | 2568929 | A/G | C6orf195 |
| 6 | rs9462671 | 189 | 8.74E-05 | 41200288 | C/T | LOC100131658 |
| 6 | rs9402592 | 11 | 3.20E-06 | 134761760 | A/G | LOC442261 |
| 6 | rs535492 | 194 | 8.95E-05 | 124905661 | A/G | NKAIN2 |
| 6 | rs505982 | 36 | 8.93E-06 | 167095386 | A/G | RPS6KA2 |
| 6 | rs635808 | 1 | 1.06E-07 | 167097412 | C/T | RPS6KA2 |
| 7 | rs6954657 | 86 | 3.67E-05 | 31549756 | A/G | CCDC129 |
| 7 | rs802443 | 49 | 1.44E-05 | 86125327 | G/T | GRM3 |
| 7 | rs802427 | 52 | 1.75E-05 | 86134271 | C/T | GRM3 |
| 7 | rs802450 | 54 | 1.82E-05 | 86135086 | G/T | GRM3 |
| 7 | rs11515001 | 192 | 8.82E-05 | 51984955 | C/T | LOC100131871 |
| 7 | rs2716751 | 159 | 7.15E-05 | 52031801 | C/T | LOC100131871 |
| 7 | rs2194591 | 50 | 1.54E-05 | 52033593 | C/T | LOC100131871 |
| 7 | rs10277728 | 221 | 9.99E-05 | 52056847 | G/T | LOC100131871 |
| 7 | rs9648034 | 55 | 1.87E-05 | 31499466 | C/T | NEUROD6 |
| 7 | rs802460 | 183 | 8.33E-05 | 86098752 | C/T | tcag7.1130 |
| 7 | rs6973392 | 30 | 7.35E-06 | 88898669 | C/T | ZNF804B |
| 7 | rs10233998 | 76 | 3.18E-05 | 88898867 | G/T | ZNF804B |
| 8 | rs10088937 | 75 | 3.15E-05 | 4282853 | A/C | CSMD1 |
| 8 | rs17057381 | 3 | 4.02E-07 | 27472718 | A/G | EPHX2 |
| 8 | rs16917973 | 173 | 7.60E-05 | 53642152 | A/G | FAM150A |
| 8 | rs2046315 | 12 | 3.28E-06 | 90280216 | C/T | LOC100129100 |
| 8 | rs2719268 | 115 | 5.13E-05 | 57034636 | A/G | LYN |
| 8 | rs4922199 | 34 | 8.25E-06 | 20345717 | A/C | LZTS1 |
| 8 | rs2170176 | 113 | 5.01E-05 | 20357815 | A/G | LZTS1 |
| 8 | rs10111661 | 83 | 3.54E-05 | 20380853 | A/G | LZTS1 |
| 8 | rs7463321 | 124 | 5.43E-05 | 20568101 | C/T | LZTS1 |
| 8 | rs2466189 | 160 | 7.18E-05 | 22907019 | C/T | PEBP4 |
| 8 | rs7835464 | 174 | 7.64E-05 | 134554009 | A/G | ST3GAL1 |
| 8 | rs4733829 | 41 | 9.87E-06 | 129132273 | C/T | TMEM75 |
| 8 | rs11167134 | 139 | 6.44E-05 | 143299436 | A/G | TSNARE1 |
| 8 | rs7463863 | 140 | 6.53E-05 | 143300073 | A/C | TSNARE1 |
| 8 | rs6583604 | 122 | 5.31E-05 | 143300487 | A/C | TSNARE1 |
| 9 | rs3860919 | 184 | 8.38E-05 | 85234358 | A/G | FRMD3 |
| 9 | rs2784068 | 171 | 7.52E-05 | 138769881 | G/T | LCN8 |
| 10 | rs7092707 | 207 | 9.73E-05 | 27199996 | C/T | ABI1 |
| 10 | rs11005115 | 125 | 5.49E-05 | 57357276 | A/G | LOC389970 |
| 10 | rs7474986 | 144 | 6.69E-05 | 57367129 | C/T | LOC389970 |
| 10 | rs11005118 | 146 | 6.79E-05 | 57370223 | C/T | LOC389970 |
| 10 | rs920526 | 191 | 8.81E-05 | 55724758 | A/G | PCDH15 |
| 10 | rs12780283 | 204 | 9.55E-05 | 55754939 | A/C | PCDH15 |
| 11 | rs10501902 | 71 | 2.93E-05 | 98485584 | A/G | CNTN5 |
| 11 | rs11607049 | 104 | 4.75E-05 | 672054 | C/T | DEAF1 |
| 11 | rs1895729 | 202 | 9.48E-05 | 106488938 | A/G | GUCY1A2 |
| 11 | rs1874795 | 211 | 9.78E-05 | 80407307 | C/T | LOC729790 |
| 11 | rs12808085 | 185 | 8.53E-05 | 62264986 | A/G | TTC9C |
| 12 | rs17107802 | 153 | 7.07E-05 | 68856527 | G/T | LOC100125409 |
| 12 | rs12817931 | 48 | 1.36E-05 | 44143762 | C/T | LOC100128248 |
| 12 | rs11058582 | 81 | 3.51E-05 | 125277759 | C/T | TMEM132B |
| 13 | rs9553843 | 145 | 6.74E-05 | 25966968 | C/T | CDK8 |
| 13 | rs2120751 | 200 | 9.45E-05 | 42661066 | A/T | DNAJC15 |
| 13 | rs10507519 | 131 | 6.08E-05 | 42713123 | C/T | ENOX1 |
| 14 | rs7157515 | 150 | 6.95E-05 | 53558366 | A/G | ATP5C2 |
| 14 | rs41535144 | 177 | 7.98E-05 | 53626570 | C/T | ATP5C2 |
| 14 | rs1957636 | 199 | 9.44E-05 | 53629768 | A/G | ATP5C2 |
| 14 | rs12588241 | 166 | 7.44E-05 | 53655593 | C/T | ATP5C2 |
| 14 | rs17710085 | 167 | 7.44E-05 | 53660093 | C/G | ATP5C2 |
| 14 | rs811140 | 117 | 5.22E-05 | 53704752 | A/G | ATP5C2 |
| 14 | rs12887156 | 163 | 7.39E-05 | 53710067 | C/T | ATP5C2 |
| 14 | rs1953743 | 8 | 1.98E-06 | 53722229 | C/T | ATP5C2 |
| 14 | rs8012357 | 64 | 2.51E-05 | 53731647 | C/T | ATP5C2 |
| 14 | rs1007141 | 175 | 7.92E-05 | 53854287 | A/G | ATP5C2 |
| 14 | rs8009160 | 57 | 2.09E-05 | 53729372 | C/G | CDKN3 |
| 14 | rs1541164 | 58 | 2.09E-05 | 53730165 | G/T | CDKN3 |
| 14 | rs8011813 | 59 | 2.10E-05 | 53731365 | C/T | CDKN3 |
| 14 | rs4251591 | 92 | 3.88E-05 | 53932460 | A/G | CDKN3 |
| 14 | rs1884014 | 78 | 3.25E-05 | 53934713 | C/G | CDKN3 |
| 14 | rs4251631 | 2 | 2.13E-07 | 53945934 | C/T | CDKN3 |
| 14 | rs10162416 | 20 | 5.12E-06 | 53959163 | C/T | CNIH |
| 14 | rs3742557 | 69 | 2.91E-05 | 53960771 | A/G | CNIH |
| 14 | rs9323264 | 22 | 5.19E-06 | 53962749 | C/T | CNIH |
| 14 | rs10220437 | 32 | 7.48E-06 | 53964046 | C/T | CNIH |
| 14 | rs10162293 | 97 | 4.06E-05 | 53983610 | A/G | CNIH |
| 14 | rs10162433 | 38 | 9.34E-06 | 53985849 | A/G | CNIH |
| 14 | rs10162584 | 40 | 9.45E-06 | 53985984 | C/T | CNIH |
| 14 | rs8007293 | 31 | 7.38E-06 | 53987417 | C/T | CNIH |
| 14 | rs11850320 | 4 | 9.92E-07 | 53990173 | G/T | CNIH |
| 14 | rs7150062 | 5 | 1.15E-06 | 53997400 | A/G | CNIH |
| 14 | rs10129440 | 17 | 4.68E-06 | 54007777 | A/G | CNIH |
| 14 | rs7142716 | 47 | 1.27E-05 | 54010107 | A/C | GMFB |
| 14 | rs7143579 | 6 | 1.16E-06 | 54010435 | A/C | GMFB |
| 14 | rs17127595 | 29 | 6.61E-06 | 54015431 | A/G | GMFB |
| 14 | rs8015391 | 16 | 3.97E-06 | 54024332 | A/G | GMFB |
| 14 | rs9323267 | 37 | 9.19E-06 | 54024580 | C/T | GMFB |
| 14 | rs7148766 | 27 | 6.43E-06 | 54026791 | A/G | GMFB |
| 14 | rs7145169 | 107 | 4.78E-05 | 100826997 | A/G | LOC100128373 |
| 14 | rs1959801 | 109 | 4.82E-05 | 100839491 | A/G | LOC100128373 |
| 14 | rs3818772 | 101 | 4.57E-05 | 100942439 | C/G | LOC100128373 |
| 14 | rs9324038 | 96 | 4.02E-05 | 100944102 | A/G | LOC100128373 |
| 14 | rs4982164 | 180 | 8.20E-05 | 33860100 | C/T | LOC100128921 |
| 14 | rs10873319 | 213 | 9.87E-05 | 78476011 | A/G | NRXN3 |
| 14 | rs7142134 | 56 | 1.91E-05 | 78476657 | C/T | NRXN3 |
| 14 | rs12588364 | 208 | 9.73E-05 | 78481089 | A/G | NRXN3 |
| 14 | rs12050287 | 219 | 9.98E-05 | 78485317 | A/T | NRXN3 |
| 14 | rs11159388 | 218 | 9.97E-05 | 78487467 | A/G | NRXN3 |
| 14 | rs11159389 | 220 | 9.98E-05 | 78487659 | C/T | NRXN3 |
| 14 | rs11159390 | 182 | 8.21E-05 | 78494228 | C/G | NRXN3 |
| 14 | rs12435173 | 95 | 4.00E-05 | 96164697 | A/G | PAPOLA |
| 14 | rs1957822 | 186 | 8.64E-05 | 96183453 | A/G | PAPOLA |
| 16 | rs16966016 | 193 | 8.93E-05 | 9656587 | C/T | LOC653737 |
| 16 | rs4888966 | 23 | 5.24E-06 | 77989354 | C/G | LOC729251 |
| 16 | rs7186103 | 222 | 9.99E-05 | 55176569 | G/T | MT4 |
| 18 | rs7243066 | 39 | 9.41E-06 | 11272319 | A/G | C18orf58 |
| 18 | rs987890 | 165 | 7.39E-05 | 11283890 | A/C | C18orf58 |
| 18 | rs8094101 | 19 | 5.10E-06 | 22937821 | C/T | CHST9 |
| 18 | rs9961915 | 18 | 4.94E-06 | 22941322 | C/T | CHST9 |
| 18 | rs16944217 | 197 | 9.43E-05 | 407542 | A/G | COLEC12 |
| 18 | rs11659443 | 61 | 2.42E-05 | 20871463 | A/G | WBP2P1 |
| 19 | rs7259269 | 67 | 2.67E-05 | 22233011 | G/T | LOC100128854 |
| 19 | rs11666460 | 63 | 2.51E-05 | 22234190 | C/G | LOC100128854 |
| 19 | rs808372 | 148 | 6.90E-05 | 22262208 | A/G | LOC100130518 |
| 19 | rs9304996 | 70 | 2.93E-05 | 22278375 | A/G | LOC100130518 |
| 19 | rs11668269 | 79 | 3.48E-05 | 22305800 | A/G | LOC100130518 |
| 19 | rs1808668 | 120 | 5.29E-05 | 22310497 | C/T | LOC100130518 |
| 19 | rs1865075 | 25 | 6.01E-06 | 22346694 | A/G | LOC342994 |
| 19 | rs10405102 | 73 | 3.02E-05 | 58262679 | C/T | ZNF160 |
| 19 | rs9304994 | 100 | 4.56E-05 | 22064583 | A/G | ZNF257 |
| 19 | rs1036235 | 108 | 4.78E-05 | 22071381 | A/G | ZNF257 |
| 19 | rs1019938 | 106 | 4.77E-05 | 22072794 | A/T | ZNF257 |
| 19 | rs1978718 | 156 | 7.14E-05 | 22084966 | A/G | ZNF257 |
| 19 | rs987711 | 157 | 7.14E-05 | 22091847 | G/T | ZNF257 |
| 19 | rs8100438 | 158 | 7.14E-05 | 22092843 | C/T | ZNF257 |
| 19 | rs452707 | 162 | 7.31E-05 | 22095985 | A/G | ZNF257 |
| 19 | rs11671804 | 217 | 9.97E-05 | 22383056 | C/T | ZNF98 |
| 19 | rs10414591 | 26 | 6.21E-06 | 22391962 | A/G | ZNF98 |
| 19 | rs10404998 | 13 | 3.30E-06 | 22400214 | C/T | ZNF98 |
| 19 | rs10405295 | 15 | 3.89E-06 | 22400396 | C/T | ZNF98 |
| 20 | rs2010809 | 91 | 3.88E-05 | 41425015 | A/G | PPIAL |
| 20 | rs501908 | 129 | 5.95E-05 | 47589174 | A/G | PTGIS |
| 20 | rs6011002 | 14 | 3.35E-06 | 61768246 | A/G | RTEL1 |
| 21 | rs7282843 | 45 | 1.24E-05 | 29397097 | A/G | C21orf7 |
| 21 | rs2832191 | 24 | 5.30E-06 | 29411171 | A/C | C21orf7 |
| 21 | rs2832194 | 65 | 2.52E-05 | 29416121 | C/T | C21orf7 |
| 21 | rs2832236 | 201 | 9.48E-05 | 29469473 | A/G | C21orf7 |
| 21 | rs6518005 | 77 | 3.25E-05 | 21089555 | A/G | LOC391271 |
| 21 | rs7277526 | 105 | 4.76E-05 | 21089877 | G/T | LOC391271 |
| 21 | rs2828438 | 170 | 7.50E-05 | 23993039 | C/T | TUBAP |
| 21 | rs2828445 | 149 | 6.93E-05 | 23999375 | A/G | TUBAP |
| 21 | rs2829459 | 154 | 7.08E-05 | 24002267 | A/G | TUBAP |
| 21 | rs2828451 | 142 | 6.57E-05 | 24007371 | C/T | TUBAP |
| 21 | rs2828453 | 138 | 6.38E-05 | 24010012 | A/G | TUBAP |
| 21 | rs2828454 | 114 | 5.12E-05 | 24012953 | A/C | TUBAP |
| 22 | rs4140448 | 130 | 6.04E-05 | 26150751 | A/G | LOC100130624 |

Supplement Table 1B: SNPs with P-value ≤ 10E-5 in Meta 2

| **Chr** | **SNP** | **rank** | **P-value** | **position** | **alleles** | **gene** |
| --- | --- | --- | --- | --- | --- | --- |
| 1 | rs12759480 | 77 | 5.73E-05 | 44301744 | C/T | SLC6A9 |
| 1 | rs10493116 | 97 | 6.78E-05 | 44302941 | C/T | SLC6A9 |
| 1 | rs11210950 | 107 | 7.64E-05 | 44313874 | A/G | SLC6A9 |
| 1 | rs3936161 | 22 | 1.55E-05 | 227336163 | A/G | RHOU |
| 1 | rs9701027 | 124 | 8.61E-05 | 227337534 | C/T | RHOU |
| 1 | rs12072775 | 55 | 4.23E-05 | 227339176 | A/C | RHOU |
| 1 | rs12065106 | 119 | 8.47E-05 | 227341339 | G/T | RHOU |
| 1 | rs9287022 | 4 | 3.79E-06 | 227344972 | A/C | RHOU |
| 1 | rs9793739 | 1 | 5.27E-07 | 227352481 | A/G | RHOU |
| 1 | rs10916435 | 102 | 7.08E-05 | 227353272 | C/G | RHOU |
| 1 | rs2988738 | 30 | 2.02E-05 | 227427128 | C/T | RHOU |
| 2 | rs10496083 | 126 | 8.82E-05 | 58070008 | G/T | LOC100131953 |
| 2 | rs1118655 | 18 | 1.37E-05 | 59692516 | C/T | LOC647038 |
| 2 | rs13420455 | 121 | 8.55E-05 | 70445311 | C/T | LOC100128042 |
| 2 | rs1106855 | 99 | 6.82E-05 | 75131495 | A/G | TACR1 |
| 2 | rs11690981 | 40 | 2.84E-05 | 108982543 | A/G | EDAR |
| 2 | rs2565215 | 103 | 7.28E-05 | 137329864 | A/G | LOC648390 |
| 2 | rs10206546 | 128 | 8.85E-05 | 144837809 | A/G | GTDC1 |
| 2 | rs4072962 | 76 | 5.71E-05 | 192411091 | A/C | SDPR |
| 2 | rs7577607 | 117 | 8.35E-05 | 192414794 | A/G | SDPR |
| 3 | rs1034383 | 86 | 6.28E-05 | 46454212 | A/G | LTF |
| 3 | rs264087 | 63 | 4.68E-05 | 65750963 | A/G | MAGI1 MAGI1 |
| 3 | rs9837471 | 37 | 2.61E-05 | 107901898 | A/G | LOC728784 |
| 4 | rs4860454 | 114 | 8.20E-05 | 62723175 | A/G | LOC391656 |
| 4 | rs2193120 | 113 | 8.17E-05 | 62725570 | C/T | LOC391656 |
| 4 | rs1870526 | 69 | 5.35E-05 | 62762334 | A/C | LOC391656 |
| 4 | rs10805050 | 6 | 4.88E-06 | 73612147 | A/G | ADAMTS3 |
| 4 | rs788911 | 5 | 4.77E-06 | 73632087 | A/C | ADAMTS3 |
| 4 | rs1383934 | 2 | 1.77E-06 | 73636388 | C/T | ADAMTS3 |
| 4 | rs10000538 | 112 | 8.05E-05 | 102161506 | C/T | LOC728771 |
| 4 | rs2141145 | 88 | 6.39E-05 | 102189271 | A/T | PPP3CA |
| 4 | rs1405686 | 132 | 9.18E-05 | 102198890 | C/T | PPP3CA |
| 4 | rs7657696 | 24 | 1.61E-05 | 136050626 | A/T | LOC345016 |
| 4 | rs9998469 | 26 | 1.76E-05 | 136058632 | A/G | LOC345016 |
| 4 | rs6814825 | 52 | 3.61E-05 | 136089958 | A/G | LOC646272 |
| 4 | rs17035648 | 72 | 5.53E-05 | 158237630 | A/G | GLRB |
| 4 | rs4422461 | 78 | 5.83E-05 | 158241732 | C/T | GLRB |
| 4 | rs11100096 | 93 | 6.53E-05 | 158255560 | A/G | GLRB |
| 4 | rs10517663 | 51 | 3.60E-05 | 158324874 | G/T | GLRB |
| 4 | rs11727838 | 50 | 3.59E-05 | 158328046 | A/G | GLRB |
| 4 | rs1353581 | 82 | 6.05E-05 | 190226983 | A/G | LOC285442 |
| 5 | rs7703230 | 43 | 3.17E-05 | 131814507 | G/T | LOC441108 |
| 5 | rs2252405 | 36 | 2.54E-05 | 131819263 | A/G | LOC441108 |
| 5 | rs4958354 | 73 | 5.64E-05 | 153440236 | C/T | MFAP3 |
| 5 | rs2923175 | 109 | 7.84E-05 | 165873669 | A/G | LOC441114 |
| 6 | rs3817837 | 129 | 9.07E-05 | 16627703 | A/G | ATXN1 |
| 6 | rs6459471 | 47 | 3.40E-05 | 16630468 | A/G | ATXN1 |
| 6 | rs6459472 | 44 | 3.30E-05 | 16630586 | A/G | ATXN1 |
| 6 | rs2268329 | 56 | 4.36E-05 | 53503322 | C/T | GCLC |
| 6 | rs3799698 | 38 | 2.77E-05 | 53509862 | C/T | GCLC |
| 6 | rs3799699 | 39 | 2.81E-05 | 53509958 | C/T | GCLC |
| 6 | rs606548 | 122 | 8.57E-05 | 53510638 | C/T | GCLC |
| 6 | rs6909381 | 89 | 6.39E-05 | 104467759 | A/G | LOC100131717 |
| 6 | rs9485905 | 115 | 8.26E-05 | 104478463 | A/C | LOC100131717 |
| 6 | rs734670 | 67 | 4.83E-05 | 137850229 | A/G | IFNGR1 |
| 6 | rs16897883 | 125 | 8.65E-05 | 165782882 | A/C | PDE10A |
| 7 | rs2108258 | 3 | 1.99E-06 | 20758523 | C/T | ABCB5 |
| 7 | rs2888830 | 8 | 7.01E-06 | 90753290 | A/G | FZD1 |
| 7 | rs13438464 | 64 | 4.76E-05 | 106378328 | G/T | PIK3CG |
| 7 | rs10263050 | 57 | 4.51E-05 | 106380844 | C/T | PIK3CG |
| 7 | rs35160619 | 84 | 6.23E-05 | 109218671 | C/T | LOC646614 |
| 8 | rs4921712 | 110 | 7.92E-05 | 20314771 | G/T | LZTS1 |
| 8 | rs13264289 | 141 | 9.98E-05 | 68409409 | C/T | ARFGEF1 |
| 8 | rs7813810 | 137 | 9.52E-05 | 68416796 | C/T | ARFGEF1 |
| 8 | rs7843081 | 92 | 6.52E-05 | 107959926 | C/T | ABRA |
| 8 | rs4269515 | 75 | 5.68E-05 | 107978185 | A/G | ABRA |
| 9 | rs2120924 | 46 | 3.40E-05 | 9166176 | A/G | PTPRD |
| 9 | rs10977553 | 61 | 4.59E-05 | 9173542 | A/G | PTPRD |
| 9 | rs12346685 | 53 | 3.65E-05 | 11596128 | G/T | LOC646114 |
| 9 | rs895023 | 35 | 2.51E-05 | 27473959 | C/T | MOBKL2B |
| 9 | rs1326930 | 127 | 8.83E-05 | 86275002 | A/G | SLC28A3 |
| 9 | rs6559808 | 74 | 5.66E-05 | 86278852 | A/G | SLC28A3 |
| 9 | rs16929545 | 105 | 7.47E-05 | 116284953 | A/G | DFNB31 |
| 9 | rs945256 | 65 | 4.79E-05 | 116925845 | C/T | TNC |
| 9 | rs1041355 | 91 | 6.48E-05 | 124357482 | A/G | OR1N2 |
| 9 | rs1578425 | 70 | 5.37E-05 | 124366334 | C/T | OR1N2 |
| 9 | rs4837991 | 80 | 5.93E-05 | 124368000 | A/G | OR1L8 |
| 9 | rs4836896 | 95 | 6.57E-05 | 124368072 | C/T | OR1L8 |
| 9 | rs4836897 | 81 | 6.04E-05 | 124368297 | C/T | OR1L8 |
| 9 | rs1999183 | 94 | 6.53E-05 | 124370680 | A/G | OR1L8 |
| 10 | rs2398002 | 87 | 6.37E-05 | 10058132 | A/G | LOC644495 |
| 10 | rs10508379 | 49 | 3.52E-05 | 10074217 | C/T | LOC644495 |
| 10 | rs7093588 | 16 | 1.22E-05 | 10082831 | C/T | LOC644495 |
| 10 | rs7917801 | 90 | 6.45E-05 | 10083592 | A/T | LOC644495 |
| 10 | rs11256432 | 68 | 5.09E-05 | 10083828 | C/T | LOC644495 |
| 10 | rs2398009 | 13 | 1.17E-05 | 10085151 | A/G | LOC644495 |
| 10 | rs7914622 | 19 | 1.51E-05 | 10087083 | A/G | LOC644495 |
| 10 | rs11256437 | 66 | 4.81E-05 | 10088763 | C/T | LOC644495 |
| 10 | rs10905651 | 9 | 7.88E-06 | 10113550 | A/G | LOC644495 |
| 10 | rs12263577 | 41 | 2.91E-05 | 10117221 | A/G | LOC644495 |
| 10 | rs9730979 | 96 | 6.68E-05 | 11778438 | A/G | LOC439951 |
| 10 | rs1983893 | 62 | 4.63E-05 | 14683476 | C/T | FAM107B |
| 10 | rs11259220 | 45 | 3.39E-05 | 14691352 | A/G | FAM107B |
| 10 | rs11000400 | 123 | 8.58E-05 | 53422481 | A/C | PRKG1 |
| 10 | rs10829630 | 85 | 6.24E-05 | 131462275 | A/G | LOC100129103 |
| 10 | rs11814529 | 136 | 9.41E-05 | 131467309 | C/T | LOC100129103 |
| 10 | rs4750773 | 25 | 1.67E-05 | 131473378 | A/G | LOC100129103 |
| 10 | rs4751122 | 20 | 1.51E-05 | 131473528 | C/G | LOC100129103 |
| 10 | rs7908271 | 138 | 9.55E-05 | 131474151 | A/G | LOC100129103 |
| 11 | rs2171456 | 101 | 7.04E-05 | 7236923 | A/G | SYT9 |
| 11 | rs10839751 | 120 | 8.52E-05 | 7239747 | A/T | SYT9 |
| 11 | rs10765975 | 116 | 8.33E-05 | 12583984 | A/G | PARVA |
| 11 | rs1566245 | 135 | 9.33E-05 | 36626354 | C/T | C11orf74 |
| 11 | rs1039205 | 79 | 5.85E-05 | 36668410 | C/T | C11orf74 |
| 11 | rs10892618 | 130 | 9.10E-05 | 120073602 | A/C | GRIK4 |
| 12 | rs11051893 | 32 | 2.12E-05 | 32318855 | A/G | BICD1 |
| 12 | rs1163656 | 10 | 8.80E-06 | 79861589 | C/T | LIN7A |
| 12 | rs7979846 | 12 | 1.16E-05 | 84970692 | A/G | MGAT4C |
| 13 | rs7331759 | 111 | 7.97E-05 | 73218523 | C/T | KLF12 |
| 13 | rs11618324 | 83 | 6.21E-05 | 73223272 | A/G | KLF12 |
| 14 | rs2415769 | 106 | 7.50E-05 | 42360599 | C/T | YWHAQP |
| 14 | rs7161648 | 60 | 4.55E-05 | 94035217 | C/T | SERPINA12 |
| 15 | rs12900194 | 108 | 7.72E-05 | 32903607 | C/G | ACTC1 |
| 15 | rs1501032 | 131 | 9.14E-05 | 51092242 | A/G | LOC645693 |
| 15 | rs1392857 | 100 | 6.95E-05 | 51101682 | C/T | LOC645693 |
| 15 | rs193097 | 133 | 9.27E-05 | 58134744 | A/G | LOC100128565 |
| 15 | rs1108435 | 54 | 4.10E-05 | 58137832 | C/T | LOC100128565 |
| 16 | rs17697259 | 29 | 2.02E-05 | 82305977 | C/T | CDH13 |
| 16 | rs17770142 | 28 | 1.99E-05 | 82309024 | A/C | CDH13 |
| 17 | rs4792515 | 139 | 9.86E-05 | 14418408 | C/T | HS3ST3B1 |
| 17 | rs4793009 | 140 | 9.98E-05 | 39071794 | A/G | MEOX1 |
| 17 | rs4793011 | 48 | 3.45E-05 | 39081851 | A/G | MEOX1 |
| 17 | rs1405952 | 27 | 1.79E-05 | 39109315 | C/T | MEOX1 |
| 17 | rs2741856 | 118 | 8.40E-05 | 39182365 | C/G | LOC100128016 |
| 17 | rs12150212 | 134 | 9.28E-05 | 72567515 | A/G | MGAT5B |
| 17 | rs7209877 | 15 | 1.22E-05 | 77248042 | C/G | CCDC137 |
| 17 | rs6565619 | 31 | 2.04E-05 | 77256301 | A/G | CCDC137 |
| 17 | rs6565620 | 11 | 1.07E-05 | 77268505 | C/T | HGS |
| 17 | rs11867462 | 14 | 1.17E-05 | 77286273 | A/G | MRPL12 |
| 17 | rs11868024 | 17 | 1.33E-05 | 77286571 | C/T | MRPL12 |
| 17 | rs3204270 | 7 | 5.35E-06 | 77292456 | C/T | SLC25A10 |
| 18 | rs1792684 | 104 | 7.35E-05 | 43623855 | C/T | SMAD2 |
| 19 | rs9967593 | 34 | 2.23E-05 | 58162248 | C/T | ZNF816A |
| 19 | rs1650966 | 33 | 2.22E-05 | 58166272 | C/T | ZNF816A |
| 20 | rs8122688 | 58 | 4.52E-05 | 604898 | A/G | SCRT2 |
| 20 | rs191705 | 23 | 1.56E-05 | 12860855 | A/G | PA2G4P2 |
| 20 | rs6014239 | 42 | 2.98E-05 | 53017454 | C/T | DOK5 |
| 22 | rs137500 | 59 | 4.52E-05 | 31606686 | A/G | SYN3 |
| 22 | rs137515 | 98 | 6.81E-05 | 31619751 | C/T | SYN3 |
| 22 | rs10483195 | 21 | 1.52E-05 | 35626482 | C/T | NCF4 |
| 22 | rs17811365 | 71 | 5.49E-05 | 35652766 | G/T | CSF2RB |

Supplement Table 1C: SNPs with P-value ≤ 10E-5 in Meta 3

| **Chr** | **SNP** | **rank** | **P-value** | **position** | **alleles** | **gene** |
| --- | --- | --- | --- | --- | --- | --- |
| 1 | rs2332267 | 95 | 6.80E-05 | 179693533 | A/G | IER5 |
| 1 | rs3936161 | 94 | 6.76E-05 | 227336163 | A/G | RHOU |
| 1 | rs12072775 | 135 | 9.50E-05 | 227339176 | A/C | RHOU |
| 1 | rs9287022 | 21 | 1.86E-05 | 227344972 | A/C | RHOU |
| 1 | rs9793739 | 7 | 4.28E-06 | 227352481 | A/G | LOC648390 |
| 1 | rs508208 | 40 | 3.53E-05 | 233794439 | A/G | GNG4 |
| 1 | rs2774327 | 106 | 7.48E-05 | 233825193 | A/G | GNG4 |
| 2 | rs568363 | 105 | 7.35E-05 | 137370465 | A/G | OXSM |
| 2 | rs4954516 | 108 | 7.56E-05 | 137956516 | C/T | THSD7B |
| 2 | rs16842873 | 143 | 9.86E-05 | 158884584 | A/G | CCDC148 |
| 3 | rs6442772 | 129 | 9.20E-05 | 3009425 | C/T | CNTN4 |
| 3 | rs4293672 | 23 | 2.12E-05 | 25888419 | C/T | OXSM |
| 3 | rs9310784 | 83 | 6.32E-05 | 25905208 | C/T | RBMS3 |
| 3 | rs11918888 | 109 | 7.63E-05 | 30346237 | C/G | RBMS3 |
| 3 | rs7634533 | 9 | 5.14E-06 | 30347057 | A/C | RBMS3 |
| 3 | rs7625884 | 16 | 1.29E-05 | 30368947 | C/T | RBMS3 |
| 3 | rs12492037 | 29 | 2.38E-05 | 30374172 | A/G | LOC728784 |
| 3 | rs17006578 | 73 | 5.80E-05 | 70017021 | A/G | MITF |
| 3 | rs9837471 | 44 | 3.64E-05 | 107901898 | A/G | LOC100132661 |
| 3 | rs12495918 | 70 | 5.74E-05 | 125065904 | A/G | MYLK |
| 3 | rs9878902 | 68 | 5.42E-05 | 145703009 | A/T | LOC100132661 |
| 3 | rs2717389 | 110 | 7.65E-05 | 145760023 | C/T | LOC727819 |
| 4 | rs4689803 | 66 | 5.26E-05 | 7676557 | A/T | SORCS2 |
| 4 | rs886372 | 63 | 5.07E-05 | 7680151 | C/T | SORCS2 |
| 4 | rs2138099 | 55 | 4.38E-05 | 16655588 | A/T | LOC729006 |
| 4 | rs16894256 | 62 | 4.81E-05 | 16656568 | A/G | LOC729006 |
| 4 | rs9992796 | 61 | 4.72E-05 | 16657296 | A/T | LOC729006 |
| 4 | rs968773 | 134 | 9.44E-05 | 34724210 | G/T | LOC391656 |
| 4 | rs4860454 | 34 | 2.97E-05 | 62723175 | A/G | LOC391656 |
| 4 | rs2193120 | 20 | 1.77E-05 | 62725570 | C/T | LOC391656 |
| 4 | rs1870526 | 38 | 3.32E-05 | 62762334 | A/C | LOC345016 |
| 4 | rs788919 | 11 | 1.02E-05 | 73572758 | C/T | ADAMTS3 |
| 4 | rs4694123 | 15 | 1.26E-05 | 73606652 | C/T | ADAMTS3 |
| 4 | rs12511787 | 17 | 1.31E-05 | 73609239 | G/T | ADAMTS3 |
| 4 | rs10805050 | 3 | 1.68E-06 | 73612147 | A/G | ADAMTS3 |
| 4 | rs788911 | 2 | 1.45E-06 | 73632087 | A/C | ADAMTS3 |
| 4 | rs1383934 | 1 | 2.96E-07 | 73636388 | C/T | ADAMTS3 |
| 4 | rs41498549 | 104 | 7.10E-05 | 102161829 | A/G | PPP3CA |
| 4 | rs34422081 | 53 | 4.28E-05 | 102161870 | C/T | PPP3CA |
| 4 | rs2141145 | 65 | 5.25E-05 | 102189271 | A/T | PPP3CA |
| 4 | rs1405686 | 113 | 8.02E-05 | 102198890 | C/T | PPP3CA |
| 4 | rs7657696 | 72 | 5.79E-05 | 136050626 | A/T | LOC345016 |
| 4 | rs9998469 | 93 | 6.73E-05 | 136058632 | A/G | SFRP2 |
| 4 | rs1339 | 64 | 5.14E-05 | 154851013 | A/G | RNF175/TLR2 |
| 4 | rs11099896 | 14 | 1.24E-05 | 154884147 | G/T | RNF175/TLR2 |
| 4 | rs12643219 | 25 | 2.16E-05 | 154890248 | C/T | RNF175/TLR2 |
| 4 | rs4696491 | 67 | 5.33E-05 | 154899315 | C/T | RNF175/TLR2 |
| 4 | rs12639885 | 33 | 2.91E-05 | 154919824 | A/G | SFRP2 |
| 4 | rs1456391 | 132 | 9.40E-05 | 154939935 | A/G | SFRP2 |
| 4 | rs6843172 | 19 | 1.62E-05 | 154980050 | C/T | GLRB |
| 4 | rs11727838 | 27 | 2.21E-05 | 158328046 | A/G | FLT4 |
| 5 | rs6876763 | 136 | 9.57E-05 | 14294854 | A/G | TRIO |
| 5 | rs6601136 | 92 | 6.70E-05 | 180053134 | A/G | OR2AI1P |
| 5 | rs3912998 | 82 | 6.30E-05 | 180058853 | A/G | HLA-DOA |
| 6 | rs6936620 | 75 | 5.88E-05 | 33092429 | A/G | HLA-DOA |
| 6 | rs6457702 | 56 | 4.53E-05 | 33096027 | G/T | KHDRBS2 |
| 6 | rs9351784 | 90 | 6.62E-05 | 63129783 | A/G | LOC728614 |
| 6 | rs1915823 | 49 | 3.94E-05 | 115070464 | G/T | LOC728614 |
| 6 | rs9481463 | 18 | 1.42E-05 | 115073292 | A/G | LOC100129634 |
| 7 | rs17203026 | 138 | 9.73E-05 | 9200889 | C/T | LOC100129634 |
| 7 | rs1529235 | 124 | 8.89E-05 | 9210866 | G/T | LOC100129634 |
| 7 | rs10237920 | 30 | 2.44E-05 | 9212099 | A/C | STK31 |
| 7 | rs2108258 | 41 | 3.58E-05 | 20758523 | C/T | ABCB5 |
| 7 | rs6977587 | 130 | 9.22E-05 | 24148468 | A/G | STK31 |
| 7 | rs1494192 | 77 | 6.04E-05 | 24153338 | G/T | HOXA9 |
| 7 | rs11773804 | 125 | 9.00E-05 | 27173213 | G/T | HOXA9 |
| 7 | rs2237336 | 89 | 6.56E-05 | 27174543 | C/T | FZD1 |
| 7 | rs6949451 | 50 | 3.98E-05 | 27181566 | C/T | HOXA10 |
| 7 | rs2888830 | 6 | 4.11E-06 | 90753290 | A/G | LOC644727 |
| 7 | rs988580 | 45 | 3.75E-05 | 111582366 | C/T | DOCK4 |
| 8 | rs2045637 | 121 | 8.56E-05 | 2963188 | A/G | CSMD1 |
| 8 | rs7822058 | 140 | 9.79E-05 | 53847414 | C/T | ABRA |
| 8 | rs6990907 | 144 | 9.87E-05 | 68316411 | A/G | ARFGEF1 |
| 8 | rs7818520 | 114 | 8.03E-05 | 68387351 | A/G | ARFGEF1 |
| 8 | rs13264289 | 85 | 6.36E-05 | 68409409 | C/T | ARFGEF1 |
| 8 | rs7813810 | 101 | 7.06E-05 | 68416796 | C/T | ARFGEF1 |
| 8 | rs7843081 | 8 | 4.34E-06 | 107959926 | C/T | ABRA |
| 8 | rs4269515 | 5 | 3.80E-06 | 107978185 | A/G | ABRA |
| 8 | rs11992975 | 54 | 4.33E-05 | 107985622 | A/G | TRPS1 |
| 8 | rs6982767 | 128 | 9.17E-05 | 116996878 | A/G | LOC646114 |
| 9 | rs2120924 | 84 | 6.34E-05 | 9166176 | A/G | PTPRD |
| 9 | rs12346685 | 100 | 6.99E-05 | 11596128 | G/T | OR1N2 |
| 9 | rs7020345 | 71 | 5.76E-05 | 100208398 | A/G | GABBR2 |
| 9 | rs16916623 | 88 | 6.44E-05 | 113861389 | A/G | SUSD1 |
| 9 | rs1578425 | 123 | 8.80E-05 | 124366334 | C/T | LOC338591 |
| 9 | rs4837991 | 60 | 4.72E-05 | 124368000 | A/G | OR1L8 |
| 9 | rs4836896 | 139 | 9.76E-05 | 124368072 | C/T | OR1L8 |
| 9 | rs4836897 | 127 | 9.06E-05 | 124368297 | C/T | OR1L8 |
| 9 | rs1999183 | 80 | 6.20E-05 | 124370680 | A/G | OR1L8 |
| 10 | rs4457655 | 142 | 9.85E-05 | 8966210 | C/T | GVIN1 |
| 10 | rs7918278 | 122 | 8.79E-05 | 11239880 | C/T | CUGBP2 |
| 10 | rs596417 | 10 | 9.97E-06 | 11258121 | C/T | CUGBP2 |
| 10 | rs10999746 | 131 | 9.35E-05 | 72677930 | A/G | UNC5B |
| 11 | rs937854 | 99 | 6.87E-05 | 6725977 | A/G | ODZ4 |
| 11 | rs2156215 | 39 | 3.45E-05 | 78503111 | G/T | LOC100125409 |
| 11 | rs7925879 | 107 | 7.50E-05 | 124245901 | A/G | ROBO3 |
| 12 | rs17107802 | 102 | 7.07E-05 | 68856527 | G/T | LIN7A |
| 12 | rs1163656 | 13 | 1.20E-05 | 79861589 | C/T | LOC401725 |
| 12 | rs1482405 | 141 | 9.82E-05 | 83189193 | A/G | DNAJC15 |
| 13 | rs912439 | 133 | 9.40E-05 | 42674703 | A/C | DNAJC15 |
| 13 | rs12873067 | 69 | 5.64E-05 | 42683268 | A/G | LOC728755 |
| 13 | rs1037022 | 91 | 6.67E-05 | 42685647 | C/T | ENOX1 |
| 13 | rs1044753 | 26 | 2.19E-05 | 42686031 | A/G | ENOX1 |
| 13 | rs703206 | 24 | 2.16E-05 | 42686036 | G/T | ENOX1 |
| 14 | rs11160621 | 47 | 3.86E-05 | 27297995 | A/G | LOC100128921 |
| 14 | rs4982164 | 116 | 8.20E-05 | 33860100 | C/T | CNIH |
| 14 | rs4251591 | 51 | 4.04E-05 | 53932460 | A/G | CDKN3 |
| 14 | rs1884014 | 98 | 6.85E-05 | 53934713 | C/G | CDKN3 |
| 14 | rs4251631 | 4 | 1.80E-06 | 53945934 | C/T | CDKN3 |
| 14 | rs8007293 | 115 | 8.17E-05 | 53987417 | C/T | FOXB1 |
| 14 | rs8015391 | 31 | 2.87E-05 | 54024332 | A/G | GMFB |
| 15 | rs335777 | 28 | 2.37E-05 | 58133159 | A/G | LOC100128565 |
| 15 | rs1108435 | 12 | 1.20E-05 | 58137832 | C/T | GRIN2A |
| 16 | rs3852751 | 32 | 2.87E-05 | 10255152 | A/C | VN1R3 |
| 16 | rs6565290 | 48 | 3.90E-05 | 31772439 | C/T | VN1R3 |
| 16 | rs7200162 | 58 | 4.65E-05 | 31789206 | C/T | ZNF267 |
| 16 | rs7203345 | 81 | 6.30E-05 | 31799737 | C/T | ZNF267 |
| 16 | rs4889517 | 126 | 9.04E-05 | 31812856 | G/T | ZNF267 |
| 16 | rs12444740 | 97 | 6.84E-05 | 31845097 | A/C | ZNF267 |
| 16 | rs1506901 | 112 | 7.96E-05 | 31851582 | C/T | ZNF267 |
| 16 | rs8064209 | 137 | 9.69E-05 | 31855262 | C/T | LOC100128016 |
| 16 | rs28377725 | 52 | 4.24E-05 | 32069996 | NA | Ambiguous |
| 17 | rs2741856 | 74 | 5.85E-05 | 39182365 | C/G | MRPL12 |
| 17 | rs7209877 | 79 | 6.19E-05 | 77248042 | C/G | CCDC137 |
| 17 | rs6565620 | 42 | 3.59E-05 | 77268505 | C/T | HGS |
| 17 | rs11867462 | 78 | 6.05E-05 | 77286273 | A/G | MRPL12 |
| 17 | rs11868024 | 96 | 6.80E-05 | 77286571 | C/T | LOC342784 |
| 17 | rs3204270 | 117 | 8.30E-05 | 77292456 | C/T | SLC25A10 |
| 18 | rs11081195 | 37 | 3.26E-05 | 5441640 | C/T | EPB41L3 |
| 18 | rs472591 | 43 | 3.62E-05 | 38737620 | C/T | RIT2 |
| 18 | rs6507872 | 111 | 7.86E-05 | 44642442 | C/T | KIAA0427 |
| 18 | rs8091995 | 86 | 6.41E-05 | 44643067 | G/T | KIAA0427 |
| 18 | rs9953008 | 118 | 8.32E-05 | 56113961 | A/G | LOC342784 |
| 18 | rs17773430 | 120 | 8.50E-05 | 56114097 | C/T | LOC100130084 |
| 19 | rs1052371 | 119 | 8.47E-05 | 7063593 | C/T | INSR |
| 19 | rs4804378 | 36 | 3.25E-05 | 8907876 | G/T | MUC16 |
| 19 | rs2288421 | 76 | 5.96E-05 | 58263118 | A/G | ZNF160 |
| 19 | rs12986307 | 22 | 2.09E-05 | 62211278 | C/T | PA2G4P2 |
| 20 | rs6074520 | 87 | 6.41E-05 | 12853188 | C/T | PA2G4P2 |
| 20 | rs191705 | 57 | 4.61E-05 | 12860855 | A/G | DOK5 |
| 20 | rs6126313 | 59 | 4.67E-05 | 49760759 | C/T | ATP9A |
| 20 | rs6014239 | 35 | 2.98E-05 | 53017454 | C/T | TUBAP |
| 21 | rs8133357 | 46 | 3.85E-05 | 21439390 | C/T | NCAM2 |
| 21 | rs2829459 | 103 | 7.08E-05 | 24002267 | A/G | TUBAP |

**Supplement Table 3: Common SNPs/regions across meta-analyses ^*^**

| **SNP** | **Chr** | **Position** | **Gene** | **P-value**  **(Meta 1)** | **P-value**  **(Meta 2)** | **P-value**  **(Meta 3)** |
| --- | --- | --- | --- | --- | --- | --- |
| rs3936161 | 1 | 227336163 | RHOU | NA | 1.55E-05 | 6.76E-05 |
| rs9701027 | 1 | 227337534 | RHOU | NA | 8.61E-05 | NA |
| rs12072775 | 1 | 227339176 | RHOU | NA | 4.23E-05 | 9.50E-05 |
| rs12065106 | 1 | 227341339 | RHOU | NA | 8.47E-05 | NA |
| rs9287022 | 1 | 227344972 | RHOU | NA | 3.79E-06 | 1.86E-05 |
| rs9793739 | 1 | 227352481 | RHOU | NA | 5.27E-07 | 4.28E-06 |
| rs10916435 | 1 | 227353272 | RHOU | NA | 7.08E-05 | NA |
| rs2988738 | 1 | 227427128 | RHOU | NA | 2.02E-05 | NA |
| rs508208 | 1 | 233794439 | GNG4 | 1.66E-05 | NA | 3.53E-05 |
| rs2774327 | 1 | 233825193 | GNG4 | NA | NA | 7.48E-05 |
| rs9837471 | 3 | 107901898 | LOC728784 | NA | 2.61E-05 | 3.64E-05 |
| rs4860454 | 4 | 62723175 | LOC391656 | NA | 8.20E-05 | 2.97E-05 |
| rs2193120 | 4 | 62725570 | LOC391656 | NA | 8.17E-05 | 1.77E-05 |
| rs1870526 | 4 | 62762334 | LOC391656 | NA | 5.35E-05 | 3.32E-05 |
| rs788919 | 4 | 73572758 | ADAMTS3 | NA | NA | 1.02E-05 |
| rs4694123 | 4 | 73606652 | ADAMTS3 | NA | NA | 1.26E-05 |
| rs12511787 | 4 | 73609239 | ADAMTS3 | NA | NA | 1.31E-05 |
| rs10805050 | 4 | 73612147 | ADAMTS3 | NA | 4.88E-06 | 1.68E-06 |
| rs788911 | 4 | 73632087 | ADAMTS3 | NA | 4.77E-06 | 1.45E-06 |
| rs1383934 | 4 | 73636388 | ADAMTS3 | NA | 1.77E-06 | 2.96E-07 |
| rs10000538 | 4 | 102161506 | PPP3CA | NA | 8.05E-05 | NA |
| rs41498549 | 4 | 102161829 | PPP3CA | NA | NA | 7.10E-05 |
| rs34422081 | 4 | 102161870 | PPP3CA | NA | NA | 4.28E-05 |
| rs2141145 | 4 | 102189271 | PPP3CA | NA | 6.39E-05 | 5.25E-05 |
| rs1405686 | 4 | 102198890 | PPP3CA | NA | 9.18E-05 | 8.02E-05 |
| rs7657696 | 4 | 136050626 | LOC345016 | NA | 1.61E-05 | 5.79E-05 |
| rs9998469 | 4 | 136058632 | LOC345016 | NA | 1.76E-05 | 6.73E-05 |
| rs11727838 | 4 | 158328046 | GLRB | NA | 3.59E-05 | 2.21E-05 |
| rs7703230 | 5 | 131814507 | IRF1 | NA | 3.17E-05 | NA |
| rs2252405 | 5 | 131819263 | IRF1 | NA | 2.54E-05 | NA |
| rs739718 | 5 | 131900972 | IRF1 | 8.20E-05 | NA | NA |
| rs2108258 | 7 | 20758523 | ABCB5 | NA | 1.99E-06 | 3.58E-05 |
| rs2888830 | 7 | 90753290 | FZD1 | NA | 7.01E-06 | 4.11E-06 |
| rs4921712 | 8 | 20314771 | LZTS1 | NA | 7.92E-05 | NA |
| rs4922199 | 8 | 20345717 | LZTS1 | 8.25E-06 | NA | NA |
| rs2170176 | 8 | 20357815 | LZTS1 | 5.01E-05 | NA | NA |
| rs10111661 | 8 | 20380853 | LZTS1 | 3.54E-05 | NA | NA |
| rs6990907 | 8 | 68316411 | ARFGEF1 | NA | NA | 9.87E-05 |
| rs7818520 | 8 | 68387351 | ARFGEF1 | NA | NA | 8.03E-05 |
| rs13264289 | 8 | 68409409 | ARFGEF1 | NA | 9.98E-05 | 6.36E-05 |
| rs7813810 | 8 | 68416796 | ARFGEF1 | NA | 9.52E-05 | 7.06E-05 |
| rs7843081 | 8 | 107959926 | ABRA | NA | 6.52E-05 | 4.34E-06 |
| rs4269515 | 8 | 107978185 | ABRA | NA | 5.68E-05 | 3.80E-06 |
| rs11992975 | 8 | 107985622 | ABRA | NA | NA | 4.33E-05 |
| rs2120924 | 9 | 9166176 | PTPRD | NA | 3.40E-05 | 6.34E-05 |
| rs12346685 | 9 | 11596128 | LOC646114 | NA | 3.65E-05 | 6.99E-05 |
| rs1578425 | 9 | 124366334 | OR1N2 | NA | 5.37E-05 | 8.80E-05 |
| rs4837991 | 9 | 124368000 | OR1L8 | NA | 5.93E-05 | 4.72E-05 |
| rs4836896 | 9 | 124368072 | OR1L8 | NA | 6.57E-05 | 9.76E-05 |
| rs4836897 | 9 | 124368297 | OR1L8 | NA | 6.04E-05 | 9.06E-05 |
| rs1999183 | 9 | 124370680 | OR1L8 | NA | 6.53E-05 | 6.20E-05 |
| rs1163656 | 12 | 79861589 | LIN7A | NA | 8.80E-06 | 1.20E-05 |
| rs2120751 | 13 | 42661066 | ENOX1 | 9.45E-05 | NA | NA |
| rs912439 | 13 | 42674703 | ENOX1 | NA | NA | 9.40E-05 |
| rs12873067 | 13 | 42683268 | ENOX1 | NA | NA | 5.64E-05 |
| rs1037022 | 13 | 42685647 | ENOX1 | NA | NA | 6.67E-05 |
| rs1044753 | 13 | 42686031 | ENOX1 | NA | NA | 2.19E-05 |
| rs703206 | 13 | 42686036 | ENOX1 | NA | NA | 2.16E-05 |
| rs10507519 | 13 | 42713123 | ENOX1 | 6.08E-05 | NA | NA |
| rs4251591 | 14 | 53932460 | CDKN3/CNIH/GMFB | 3.88E-05 | NA | 4.04E-05 |
| rs1884014 | 14 | 53934713 | CDKN3/CNIH/GMFB | 3.25E-05 | NA | 6.85E-05 |
| rs4251631 | 14 | 53945934 | CDKN3/CNIH/GMFB | 2.13E-07 | NA | 1.80E-06 |
| rs8007293 | 14 | 53987417 | CDKN3/CNIH/GMFB | 7.38E-06 | NA | 8.17E-05 |
| rs8015391 | 14 | 54024332 | CDKN3/CNIH/GMFB | 3.97E-06 | NA | 2.87E-05 |
| rs335777 | 15 | 58133159 | ANXA2 | NA | NA | 2.37E-05 |
| rs193097 | 15 | 58134744 | ANXA2 | NA | 9.27E-05 | NA |
| rs1108435 | 15 | 58137832 | ANXA2 | NA | 4.10E-05 | 1.20E-05 |
| rs2741856 | 17 | 39182365 | LOC100128016 | NA | 8.40E-05 | 5.85E-05 |
| rs7209877 | 17 | 77248042 | CCDC137 | NA | 1.22E-05 | 6.19E-05 |
| rs6565620 | 17 | 77268505 | HGS | NA | 1.07E-05 | 3.59E-05 |
| rs11867462 | 17 | 77286273 | MRPL12 | NA | 1.17E-05 | 6.05E-05 |
| rs11868024 | 17 | 77286571 | MRPL12 | NA | 1.33E-05 | 6.80E-05 |
| rs3204270 | 17 | 77292456 | SLC25A10 | NA | 5.35E-06 | 8.30E-05 |
| rs9967593 | 19 | 58162248 | ZNF160/816A/702P | NA | 2.23E-05 | NA |
| rs1650966 | 19 | 58166272 | ZNF160/816A/702P | NA | 2.22E-05 | NA |
| rs10405102 | 19 | 58262679 | ZNF160/816A/702P | 3.02E-05 | NA | NA |
| rs191705 | 20 | 12860855 | PA2G4P2 | NA | 1.56E-05 | 4.61E-05 |

* summarize SNPs or nearby regions (within 100kb) containing SNPs with P-value ≤ 10E-5 and presented in at least two meta-analyses;
